# Treatment of advanced atherosclerotic mice with ABT-263 reduced indices of plaque stability and increased mortality

**DOI:** 10.1172/jci.insight.173863

**Published:** 2024-01-23

**Authors:** Santosh Karnewar, Vaishnavi Karnewar, Laura S. Shankman, Gary K. Owens

**Affiliations:** Robert M. Berne Cardiovascular Research Center, University of Virginia-School of Medicine, Charlottesville, Virginia, USA.

**Keywords:** Vascular Biology, Atherosclerosis

## Abstract

The use of senolytic agents to remove senescent cells from atherosclerotic lesions is controversial. A common limitation of previous studies is the failure to rigorously define the effects of senolytic agent ABT-263 (Navitoclax) on smooth muscle cells (SMC) despite studies claiming that these cells are the major source of senescent cells. Moreover, there are no studies on the effect of ABT-263 on endothelial cells (EC), which — along with SMC — comprise 90% of α-smooth muscle actin^+^ (α-SMA^+^) myofibroblast-like cells in the protective fibrous cap. Here we tested the hypothesis that treatment of advanced atherosclerotic mice with ABT-263 will reduce lesion size and increase plaque stability. SMC (Myh11-CreER^T2^-eYFP) and EC (Cdh5-CreER^T2^-eYFP) lineage tracing *Apoe^–/–^* mice were fed a western diet (WD) for 18 weeks, followed by ABT-263 at 100 mg/kg/bw for 6 weeks or 50 mg/kg/bw for 9 weeks. ABT-263 treatment did not change lesion size or lumen area of the brachiocephalic artery (BCA). However, ABT-263 treatment reduced SMC by 90% and increased EC contributions to lesions via EC-to-mesenchymal transition (EndoMT) by 60%. ABT-263 treatment also reduced α-SMA^+^ fibrous cap thickness by 60% and was associated with a > 50% mortality rate. Taken together, ABT-263 treatment of WD-fed *Apoe^–/–^* mice with advanced lesions resulted in multiple detrimental changes, including reduced indices of stability and increased mortality.

## Abstract 2

<p>Treatment of Western diet-fed *Apoe*^-/-^ mice with the senolytic agent ABT-263 results in multiple detrimental changes, including reduced indices of plaque stability and increased mortality.</p>

## Graphical abstract

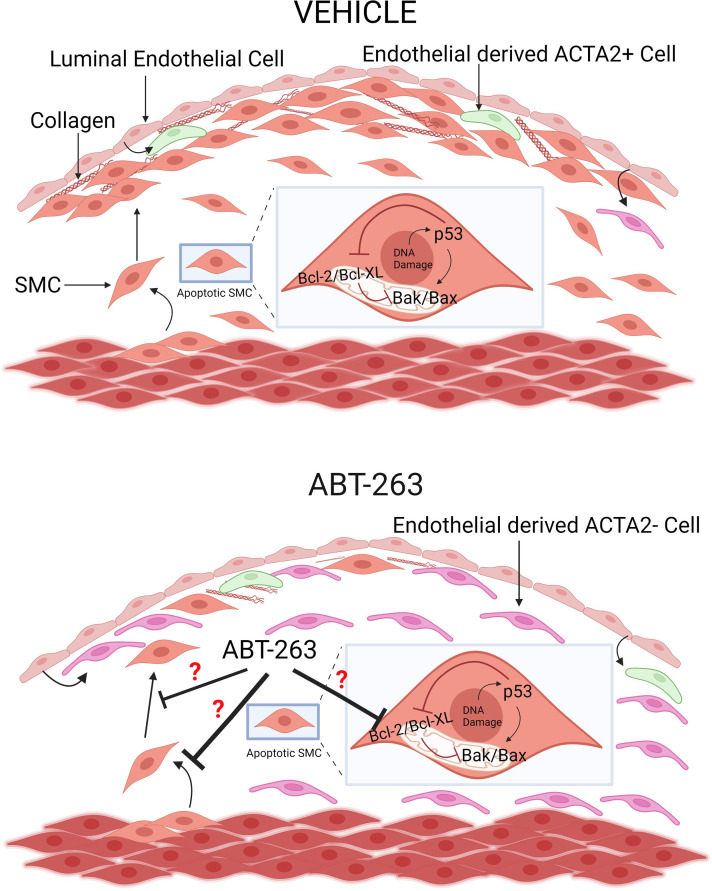

## Introduction

The rupture or erosion of advanced atherosclerotic plaques resulting in ischemic heart disease or stroke are the leading causes of death worldwide (1). Human histopathological studies have clearly established that lesions containing a thick extracellular matrix–rich (ECM-rich) fibrous cap and an abundance of α-smooth muscle actin^+^ (α-SMA^+^) versus CD68^+^ cells are less likely to rupture (2). Most investigators in the field have assumed that α-SMA^+^ lesion cells are derived from smooth muscle cells (SMC) and have a beneficial role in lesion pathogenesis by being the exclusive source of ECM-producing fibrous cap cells (3). In addition, they assumed that CD68^+^ cells are macrophages that exacerbate lesion pathogenesis by increasing inflammation and giving rise to foam cells. However, subsequent studies by our lab (4) and many others (5–8) have either refuted several key aspects of these assumptions or shown that they are overly simplistic.

First, α-SMA^+^ staining is highly unreliable for identifying SMC-derived cells in lesions, given that nearly 80% of SMC-derived lesion cells lack detectable expression of this marker and activate markers of other cell types including macrophages (4). In addition, we recently showed that 40% of α-SMA^+^ fibrous cap cells are derived from a non-SMC, including EC that have undergone EC-to-mesenchymal transition (EndoMT) and macrophages undergoing macrophage-to-myofibroblast transition (MMT) in advanced BCA lesions from *Apoe^–/–^* and *Ldlr*-deficient mice fed a western diet (WD) for 18 weeks (9). Second, there are qualitative differences in plaque stability depending on the source of α-SMA^+^ fibrous cap cells, with increased EndoMT and MMT only being able to transiently compensate for the loss of SMC investment into lesions induced by SMC KO of *Pdgfβ*r or lethal irradiation (9). Third, there is compelling evidence showing that each of the major cell types involved in atherosclerosis, including macrophages (10), T cells (11), B cells (12), neutrophils (13), endothelial cells (EC) (9), and SMC (4, 14–16), can have beneficial or detrimental effects on lesional development or late-stage pathogenesis depending on the nature of their phenotypic transitions (17). For example, using a combination of SMC lineage tracing, SMC-specific KO of *Klf4* or *Oct4*, and single-cell RNA-Seq (scRNA-Seq) analysis of lesions, we showed that SMC can have a beneficial or detrimental role in lesion pathogenesis (15). Results with SMC KO of *Klf4* were particularly intriguing, since this resulted in what would be the ideal therapeutic outcome in patients at risk for development of atherosclerosis. Specifically, SMC-*Klf4*–KO lesions were 50% smaller and exhibited features associated with increased plaque stability, including a 2-fold increase in the thickness of the ECM-rich α-SMA^+^ fibrous cap(4). *Klf4* was subsequently identified as a coronary artery disease genome-wide association study (GWAS) variant (18). Moreover, our lab recently showed that loss of *Klf4* in SMC resulted in a marked reduction of mRNA transcripts associated with cellular senescence and inflammation, including > 40 putative *KLF4* target genes previously identified as coronary artery disease GWAS variants. Taken together, results suggest that the beneficial effects of SMC *Klf4* KO in mouse models, and beneficial effects of *Klf4* being a CAD GWAS variant, are due in part to its role in enhancing cell senescence and inflammation.

Senescence is a term used to define a state of irreversible growth arrest and includes replicative senescence, or stress-induced premature senescence (SIPS) (19). Indeed, previous studies have shown that the atherosclerotic lesions in humans and mice contain relatively large numbers of senescent cells (20–22), and they claim that these senescent cells promote atherosclerosis and contribute to destabilization of plaques (23). However, the origins and functional properties of these senescent cells are uncertain, since they relied on methods that eliminated “senescent cells” based on expression of a single marker gene, which is not specific for senescent cells, and/or relied on 1 or 2 traditional, albeit unreliable, markers to identify SMC-, EC- and macrophage-derived cells within lesions (23, 24). For example, when Childs et al. (25) reduced senescent cells using a ganciclovir-dependent (GCV-dependent) *p16*-driven thymidine kinase genetic approach to induce death of p16^Ink4a+^ cells, they discovered reduced Sudan-IV^+^ plaque burden and increased fibrous cap thickness of atherosclerotic lesions within the descending aorta of *Ldlr^–/–^* female mice. This same study showed that treatment of *Ldlr^–/–^* female mice at a dosage of 100 mg/kg/body weight (bw) of the senolytic drug ABT-263 (Navitoclax) throughout 88 days of high-fat diet feeding reduced the Sudan-IV^+^ area of the aorta(25). In addition, a more recent study by Childs et al. (26) showed that aortic arch lesions of Myh11-CreER^T2^ TdTomato *Ldlr^–/–^* male mice fed a WD for 14 weeks, followed by 9 weeks of a low-fat diet (LFD) and treatment with ABT-263, had a reduced proportion of tdTomato^+^ cells that expressed the osteochondrogenic marker *Runx1* as compared with vehicle-treated mice (26). ABT-263 inhibits interactions between the antiapoptotic protein BCL-X_L_ and the proapoptotic protein BAX, thus enhancing clearance of senescent cells by reducing their capacity to evade efferocytotic clearance (27). ABT-263 disrupts *Bcl-2/Bcl-xL* interactions with prodeath proteins (e.g., Bim), leading to the initiation of apoptosis (28). Thus, Childs et al. (25, 26) interpreted their results as evidence that the beneficial effects on atherosclerosis-reduced plaque burden observed with ABT-263 treatment were the direct result of enhanced clearance of senescent cells from lesions. In recent years, these results have been challenged by studies by Martin Bennett and coworkers who showed the following (29). First, they found that “presumed” selective clearance of senescent cells using a p16-driven thymidine kinase genetic approach similar to Childs et al. (25) did not reduce Oil red O^+^ plaque burden, aortic root lesion size, fibrous cap thickness, or necrotic core area in *Apoe^–/–^* mice (males and females combined); instead, it increased apoptotic cells and induced inflammation. Second, treatment of *Apoe^–/–^* male and female mice with established lesions with 50 mg/kg/bw of ABT-263 did reduce Oil red O^+^ plaque burden and aortic root lesion size, but it did not increase cap thickness. Third, ABT-263 treatment of *Apoe^–/–^* mice did not reduce relative mRNA expression of *p16* and senescence-associated secretory phenotypes (SASPs), including *IL-6*, *IL-1*α, *Tnf-*α, *IL-18*, and *Mmp-12*, in aortic arch tissue, but it increased thrombocytopenia and reduced monocyte and leukocyte populations. Another independent study from James Kirkland’s lab showed that treatment of *Apoe^–/–^* mice with the senolytic agent Dasatinib + Quercetin did not reduce lesion size but reduced plaque calcification (30). The reasons for the disparate results of the preceding studies are unclear but may be related to male versus female sex, *Apoe^–/–^* versus *Ldlr^–/–^* mice, and differences in the senolytic drugs tested and/or dosing regimens. However, a common limitation of these previous studies was the failure to rigorously define the effects of ABT-263 on SMC, despite each study claiming that this is the major source of senescent cells. In addition, there have been no studies of the effects of ABT-263 on EC, which along with SMC, compose 90% of α-SMA^+^ myofibroblast-like cells in the protective fibrous cap. These studies also include minimal assessment of indices of plaque stability and were largely focused on assessment of aortic root lesions, which poorly replicate the histology of human lesions. There are still major uncertainties regarding the effects of senolytic therapies on the cellular composition of advanced atherosclerotic lesions, the phenotypes of the lesion cells, and overall indices of plaque stability that need to be clarified before determining their usefulness in treating patients with advanced atherosclerotic disease.

Studies herein test two hypotheses. First, we hypothesized that the beneficial effects of SMC KO of Klf4 are due in part to reduced cell senescence and an associated reduction in inflammation. Second, we hypothesized that treatment of WD-fed SMC and EC lineage tracing *Apoe^–/–^* mice with the senolytic agent ABT-263 will result in loss of senescent cells from atherosclerotic lesions, thereby reducing lesion inflammation and size as well as inducing changes consistent with increased plaque stability, including a thicker SMC- and ECM-rich α-SMA^+^ protective fibrous cap. Consistent with our first hypothesis, KO of *Klf4* in SMC in WD-fed *Apoe^–/–^* mice was associated with marked reductions in plaque size, lipid deposition, and the overall prevalence of senescent cells. However, contrary to expectations, the results of studies testing our second hypothesis showed that treatment of WD-fed *Apoe^–/–^* mice with ABT-263 had multiple detrimental effects, including inducing marked reductions in Myh11-eYFP^+^ SMC–derived cells within lesions and the fibrous cap, reduced α-SMA^+^ fibrous cap thickness, increased EndoMT, and increased mortality.

## Results

### Klf4 KO in SMC resulted in reduced overall lesion senescence.

**Figure 1:**
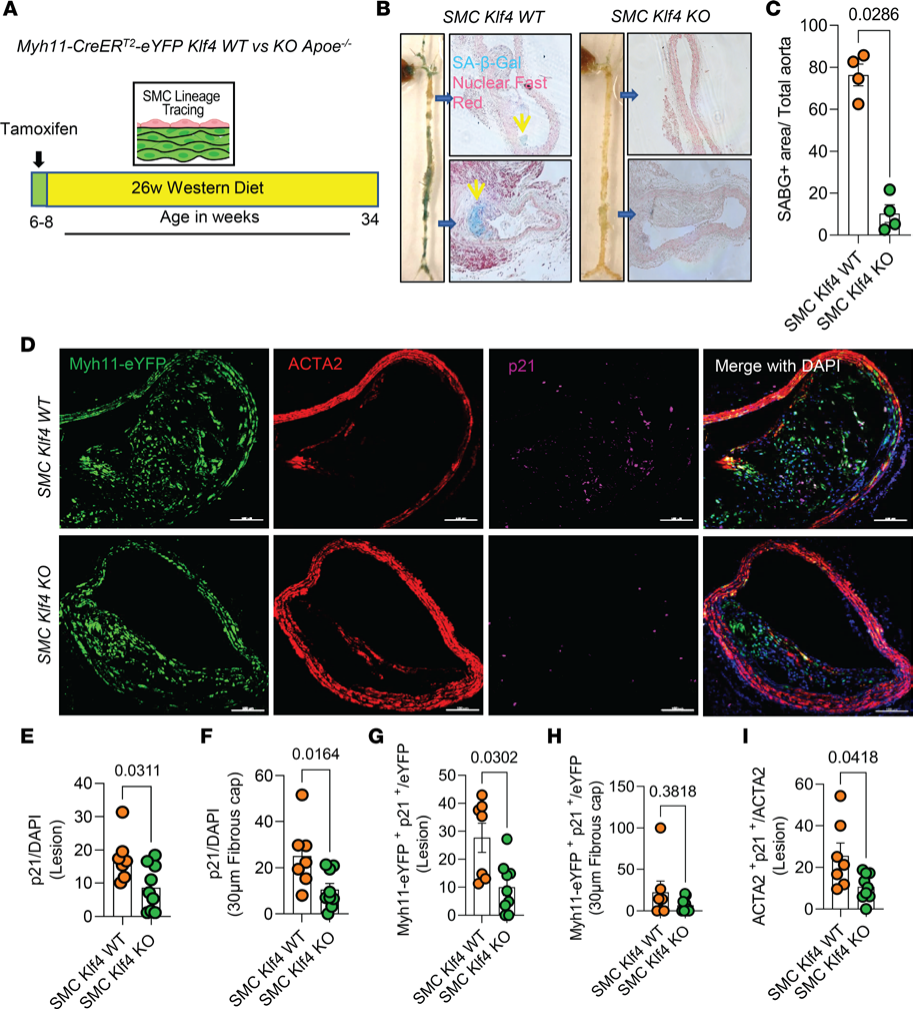
SMC-Klf4 KO resulted in a marked reduction in overall lesion senescence, including reduced expression of the prosenescence markers *p21*^+^ and SAβG^+^ cells. (**A**) Experimental design, SMC (Myh11-CreER^T2^-eYFP) lineage tracing *Klf4* WT versus KO *Apoe^–/–^* mice were injected with tamoxifen at 6–8 weeks of age and subsequently placed on western diet (WD) for 26 weeks to induce advanced atherosclerosis. (**B**) SAβG staining was performed as described in Methods on freshly isolated aortas. Thoracic and abdominal aortic sections from SAβG-stained aortas from the left images and counter stained with nuclear fast red and yellow arrows indicate the SAβG^+^ cells. Original magnification ×10. (**C**) Quantification of SAβG^+^ area of the aorta. (**D**) Representative images of costaining for eYFP (for detecting SMC), α-SMA, *p21* (a marker of senescence), and DAPI (nucleus) in advanced BCA lesions from SMC *Klf4* WT and KO animals fed a WD for 26 weeks. The confocal images show a maximum intensity projection ×20 zoom. Scale bar: 100 μm. (**E** and **F**) Quantification of the frequency of *p21^+^* (p21^+^/DAPI) senescent cells in the lesion (**E**) and fibrous cap (**F**) as a percent of total cells in the lesions. (**G** and **H**) Quantification of SMC derived *p21^+^* (eYFP^+^ p21^+^/eYFP) senescent cells in the lesion (**G**) and the fibrous cap (**H**) as a percent of total SMC. (**I**) Quantification of α-SMA^+^ senescent (α-SMA^+^ p21^+^/α-SMA) cells in the lesion as a percentage of total α-SMA^+^ cells. Mann-Whitney *U* tests were used for statistical analysis in **C** and **E**–**I**. Data are shown as mean ± SEM. Independent animals are indicated as individual dots (WT, *n* = 7, and KO, *n* = 9). The *P* values are indicated on the respective graphs.

As an initial test of our hypothesis that the multiple beneficial effects of SMC KO of Klf4 are due in part to reduced cell senescence, male SMC *Klf4* WT and KO *Apoe^–/–^* mice were fed a WD for 18 weeks (Supplemental Figure 1; supplemental material available online with this article; https://doi.org/10.1172/jci.insight.173863DS1) or 26 weeks (Figure 1A), and aortas were then stained for senescence-associated β-galactosidase (SAβG). Interestingly, SMC *Klf4* WT mouse aortas showed highly prevalent SAβG^+^ senescent cells in the brachiocephalic artery (BCA), aortic root, aortic arch, and abdominal aorta (Figure 1B). In contrast, there was almost no SAβG^+^ staining in SMC *Klf4*–KO mice (Figure 1, B and C), as seen in whole aortic preparations and in cross sections of the thoracic and abdominal aorta counterstained with nuclear fast red (Figure 1B). Similar results were found in mice fed WD for 18 weeks (Supplemental Figure 1, B and C). Loss of *Klf4* in SMC was also associated with an increase in the telomere reverse transcriptase (*Tert^+^*) antisenescence marker but with a decrease in *p16^+^* (prosenescence marker) clusters, as assessed from uniform manifold approximation and projections (UMAPs) of scRNA-Seq data sets of BCA lesions from SMC *Klf4* WT and KO mice (15) (Supplemental Figure 1, D and E). KO of *Klf4* in SMC preserved the *Tert^+^* cells in most of the clusters of lesion cells as shown by UMAP analysis (Supplemental Figure 1D). In contrast, KO of *Klf4* in SMC reduced the number of SMC-derived cells positive for the prosenescence marker *p16* (*Cdkn2a*) (Supplemental Figure 1E). Indeed, our previous study showed that *Klf4* binds to Telomeric RNA (*TERC*) in SMC of advanced atherosclerotic lesions based on ChIP assays (4). Next, cultured murine aortic SMC were transfected with siKlf4 to determine if knockdown (KD) of *Klf4* in SMC reduces the prosenescent marker *p16* directly or through crosstalk with other cell types in the lesion. Results showed that KD of *Klf4* reduced *p16* expression in SMC at both the mRNA and protein levels (Supplemental Figure 1, F and G).

**Figure 2:**
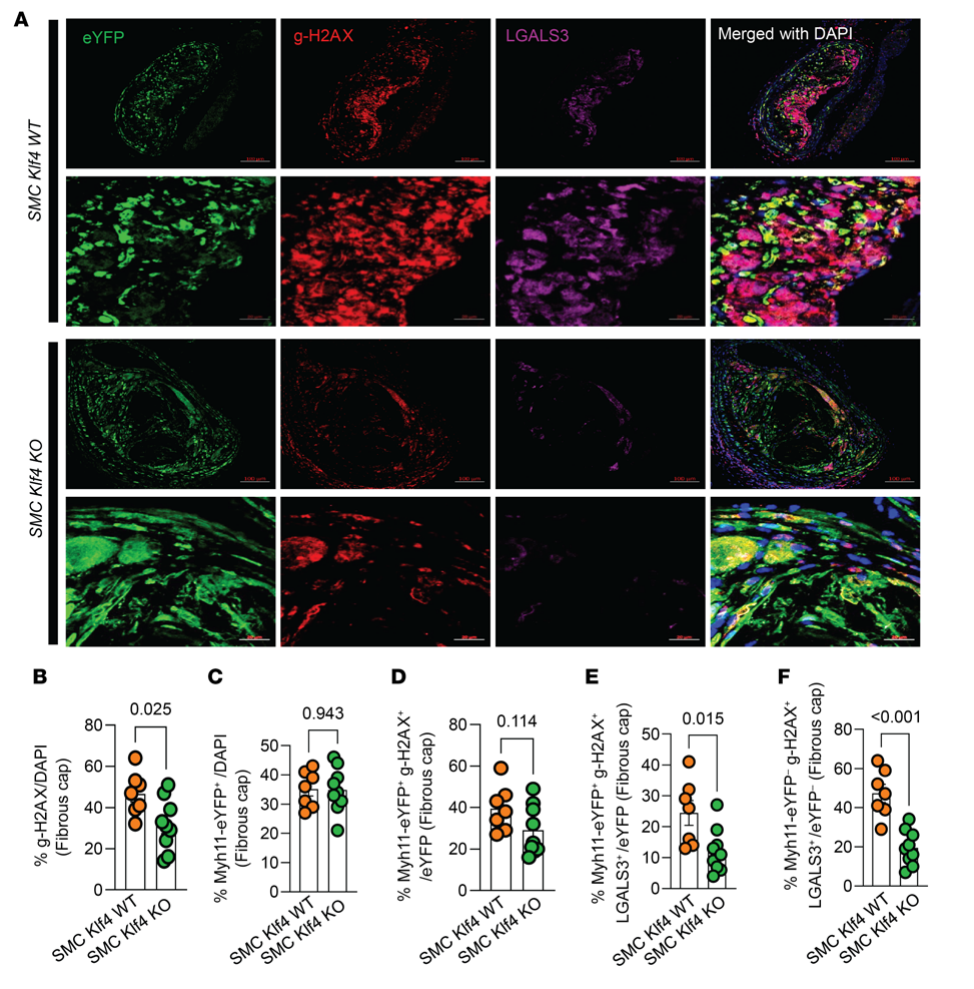
SMC-Klf4 KO resulted in a marked reduction of SMC-derived and non–SMC-derived LGALS3^+^ cells also positive for the senescence marker γ-H2AX. (**A**) Representative images of costaining for eYFP (for detecting SMC), ***γ-**H2AX* (a marker of senescence), LGALS3, and DAPI (nucleus) in advanced BCA lesions from SMC *Klf4* WT and KO animals fed WD for 26 weeks as shown in Figure 1A. The confocal images show a maximum intensity projection ×20 zoom. Scale bar: 100 μm and 20 μm (zoomed-in images region of interest). (**B**) Quantification of the frequency of H2AX^+^ (H2AX^+^/DAPI) senescent cells in the fibrous cap. (**C**) Myh11-eYFP^+^/DAPI in the fibrous cap. (**D**) SMC-derived γ-H2AX^+^ cells of all SMC (Myh11-eYFP^+^ γ-H2AX^+^/eYFP) in the fibrous cap. (**E**) Quantification of SMC-derived γ-H2AX^+^ LGALS3^+^ (Myh11-eYFP^+^ γ-H2AX^+^ LGALS3^+^/eYFP) of all SMC in the fibrous cap. (**F**) Quantification of non–SMC-derived γ-H2AX^+^ LGALS3^+^ (Myh11-Eyfp^–^ γ-H2AX^+^ LGALS3^+^/eYFP^–^) cells of all non-SMC cells in the fibrous cap. Mann-Whitney *U* tests were used for statistical analysis in **B**–**F**. Data are shown as mean ± SEM. Independent animals are indicated as individual dots (WT, *n* = 7, and KO, *n* = 9). The *P* values are indicated on the respective graphs.

A previous study from our lab showed that *Klf4* is a suppressor of SMC differentiation markers and an inhibitor of SMC proliferation by inducing *p21*^WAF1/Cip1^ expression in concert with *p53* (31). Both *p53* and *p21* are widely accepted prosenescence markers (32). Therefore, we stained BCA sections of SMC *Klf4* WT and KO mice fed a WD for 18 weeks with a p21 antibody to determine if SMC *Klf4* KO would reduce the frequency of p21^+^ senescent cells in atherosclerotic lesions (Figure 1D). As predicted, there was a reduction in p21^+^ senescent cells (p21/DAPI) in the BCA lesions and fibrous cap of SMC *Klf4*–KO versus WT control mice (Figure 1, E and F). The fraction of SMC-derived p21^+^ cells (Myh11-eYFP^+^ p21^+^/eYFP) was also reduced in the lesions but not in the fibrous cap of these same mice (Figure 1, G and H). Furthermore, the fraction of α-SMA^+^ cells that expressed the senescence marker p21 was also reduced in the lesions of SMC *Klf4*–KO versus WT control mice (Figure 1I). Furthermore, **γ-**H2AX, another common marker of cellular senescence and a sensitive marker of double-stranded DNA breaks and telomere shortening (33, 34), was also reduced in the fibrous cap of SMC *Klf4*–KO BCA as compared with their WT controls (Figure 2, A and B). However, we did not see any change in SMC (Myh11-eYFP^+^/DAPI) or SMC-derived **γ-**H2AX^+^ (Myh11-eYFP^+^ **γ-**H2AX^+^/eYFP) cells (Figure 2, C and D). Interestingly, the fraction of SMC-derived **γ-**H2AX^+^ LGALS3^+^ (Myh11-eYFP^+^ **γ-**H2AX^+^ LGALS3^+^/eYFP) cells was reduced (Figure 2E). There was also a reduction in non–SMC-derived **γ-**H2AX^+^ LGALS3^+^ (Myh11-eYFP^–^ **γ-**H2AX^+^ LGALS3^+^/eYFP^–^) cells (Figure 2F). These results indicate that SMC-derived and non–SMC-derived senescent cells were also LGALS3^+^ cells that were reduced with SMC *Klf4* KO. Importantly, these results extend our previous studies by Shankman et al. and Alencar et al., where we found that SMC-derived LGALS3^+^ cells were reduced with SMC *Klf4* KO (4) and that these SMC-derived *Lgals3*^+^ cells had transitioned to osteochondrogenic and senescence marker clusters (15). Results show that KO of *Klf4* in SMC was associated with a marked reduction in the prevalence of senescent cells within the aorta and BCA lesions of *Apoe^–/–^* mice fed WD for 18 or 26 weeks.

Taken together, the preceding results extend results of previous studies from our lab (4, 15), showing that SMC *Klf4* KO has multiple beneficial effects, including resulting in smaller lesions with a thicker α-SMA^+^ fibrous cap (4) containing reduced numbers of senescent cells (Figure 1 and Supplemental Figure 1). However, it is not clear if the reduction in senescent cells contributes causally to the beneficial effects on lesions or if the reduced number of senescent cells is secondary to loss of *Klf4* in SMC acting through other mechanisms. To distinguish these possibilities, we sought alternative approaches to reduce the prevalence of senescent cells within advanced lesions.

### Treatment of Apoe^–/–^ mice with advanced atherosclerotic lesions with the senolytic agent ABT-263 reduced the number of SMC within BCA lesions and was associated with increased EndoMT-derived lesion cells and mortality.

**Figure 3:**
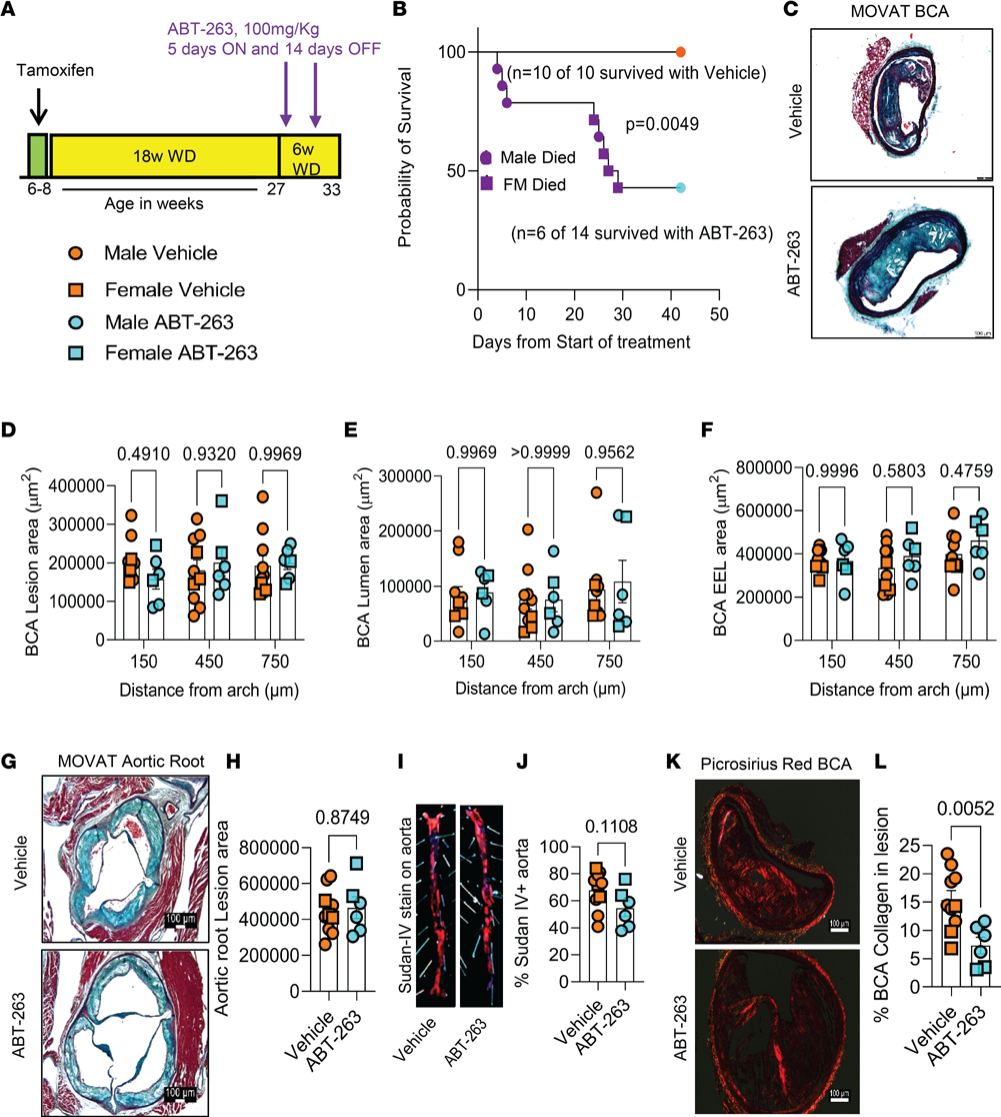
ABT-263 treatment of SMC (Myh11-CreER^T2^-eYFP) and EC (Cdh5-CreER^T2^-eYFP) lineage tracing Apoe^–/–^ mice with advanced atherosclerotic lesions had no effect on lesion size but increased mortality. (**A**) Experimental design. SMC and EC lineage tracing *Apoe^–/–^* mice were fed a WD for 18 weeks followed by ABT-263 treatment on western diet (WD) for 6 weeks. (**B**) Probability of survival (Kaplan-Meier curve). (**C**) Representative 10× images of MOVAT staining on brachiocephalic artery (BCA). Scale bar: 100 μm. (**D**) Lesion area from **C**. (**E**) Lumen area from **C**. (**F**) External elastic lamina (EEL) area from **C**, for outward remodeling. (**G**) Aortic roots stained with MOVAT. Scale bar: 100 μm. (**H**) Lesion area quantification from **G**. (**I**) Representative Sudan-IV–stained aortas from vehicle or ABT-263 treatment. (**J**) Quantification of % Sudan-IV+ aorta. (**K**) Representative 10x images of Picrosirius red staining on Brachiocephalic Artery (BCA). Scale bar: 100 μm. (**L**) Quantification of matured (red) collagen content normalized to lesion area from **K**. A repeated-measures 2-way ANOVA method was used for statistical analysis in **D**–**F**, whereas Mann-Whitney *U* tests were used for statistical analysis in **H**, **J**, and **L**. Biologically independent animals are indicated as individual dots; data are shown as mean ± SEM. A Mantel-Cox test was used for statistical analysis in **B**. The *P* values are indicated on the respective graphs.

**Figure 4:**
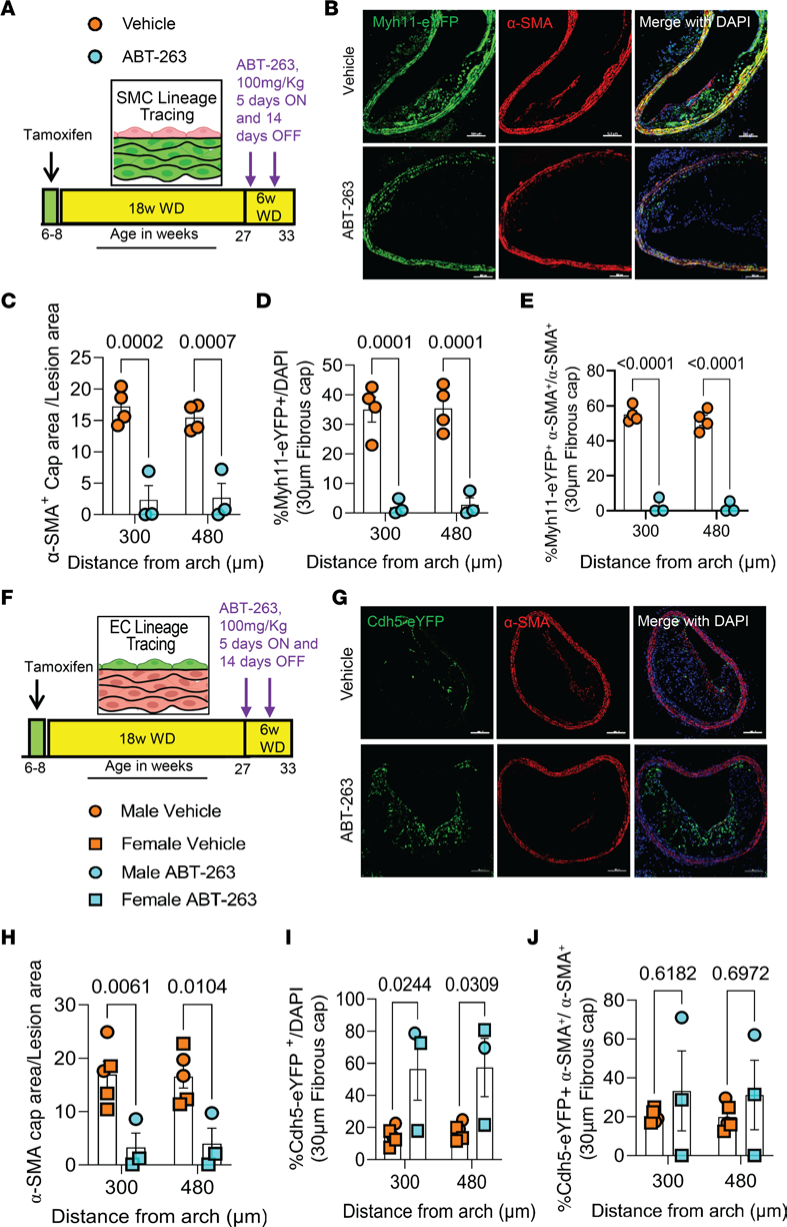
Treatment of *Apoe*^–/–^ mice with advanced atherosclerotic lesions with the senolytic agent ABT-263 was associated with a marked reduction in the number of SMC within BCA lesions but an increase in EC-derived cells undergoing EndoMT. ABT-263 treatment (100 mg/kg/bw) of SMC (Myh11-CreER^T2^-eYFP) and EC (Cdh5-CreER^T2^-eYFP) lineage tracing *Apoe*^–/–^ mice with advanced atherosclerotic lesions was associated with a marked reduction in the number of SMC within BCA lesions but an increase in EC-derived cells undergoing EndoMT. However, the latter did not result in increased investment of EC-derived α-SMA^+^ cells into the fibrous cap. (**A**) Experimental design for **B**–**E**. SMC lineage tracing *Apoe^–/–^* mice were fed a WD for 18 weeks followed by 100 mg/kg/bw ABT-263 treatment on WD for 6 weeks. (**B**) Representative confocal images of costaining for eYFP (for detecting SMC), α-SMA^+^, and DAPI in advanced BCA lesions from **A**. The confocal images show a maximum intensity projection ×20 zoom. Scale bar: 100 μm. (**C**) α-SMA^+^ cap area normalized to lesion area (α-SMA^+^ cap area/lesion area). (**D**) Quantification of the percentage of SMC-derived (Myh11-eYFP^+^/DAPI^+^) cells in the fibrous cap. (**E**) Quantification of the percentage of SMC-derived α-SMA^+^ (Myh11-eYFP^+^ α-SMA^+^/α-SMA^+^) cells in the fibrous cap. (**F**) The experimental design for **G**–**J**, EC-lineage tracing Apoe^–/–^ mice were fed a WD for 18 weeks followed by 100 mg/kg/bw ABT-263 treatment on WD for 6 weeks. (**G**) Representative confocal images of costaining for eYFP (for detecting EC), α-SMA^+^ and DAPI in advanced BCA lesions from **F**. (**H**) α-SMA^+^ Cap area normalized to lesion area. (**I**) Quantification of the percentage of EC-derived Cdh5-eYFP^+^DAPI^+^ cells in the fibrous cap. (**J**) Quantification of the percentage of EC-derived α-SMA^+^ (Cdh5-eYFP^+^α-SMA^+^/α-SMA^+^) cells in the fibrous cap. The two-way ANOVA method was used 2 statistical analyses in **C**–**E** and **H**–**J**. Biologically independent animals are indicated as individual dots. Data are shown as mean ± SEM . The *P* values are indicated on the figures.

To determine if increased clearance of senescent cells could induce beneficial changes in late-stage complex atherosclerotic lesions, SMC and EC lineage tracing *Apoe^–/–^* mice were fed a WD for 18 weeks and then treated with 3 cycles (Figure 3A) of 100 mg/kg/bw ABT-263 as used previously by Childs et al. (25). We elected to do an intervention rather than a prevention study to better model clinical scenarios in patients with advanced disease (35). However, the experiment had to be unexpectedly terminated for ethical reasons, as per our IACUC-approved animal protocol, because after 2 cycles of ABT-263, > 50% of the ABT-263–treated mice died or had to be ethically euthanized (Figure 3B). ABT-263–treated mice had no changes in BCA lesion size (Figure 3, C and D), lumen area, or outward remodeling (external elastic lamina [EEL] area) (Figure 3, E and F); no change in aortic root lesion area; and no change in plaque burden (Figure 3, G–J). However, ABT-263–treated mice showed multiple detrimental changes in lesion composition, including reduced BCA lesion collagen content (Figure 3, K and L), a reduced α-SMA^+^ fibrous cap area (Figure 4, A–C), and a reduced fraction of α-SMA^+^ or α-SMA^–^ SMC–derived cells (Myh11-eYFP^+^/DAPI) in lesions and the 30 μm fibrous cap area (Figure 4, B, D, and E, and Supplemental Figure 2, A–C). To determine if the non–SMC-derived α-SMA^+^ fibrous cap cells in this study were derived from EC, we treated EC lineage tracing mice with the same dose of ABT-263 and assessed (Figure 4F) BCA lesions for eYFP, α-SMA, and DAPI (Figure 4G). The ABT-263 treatment also reduced the α-SMA^+^ cap area and the fraction of α-SMA^+^ (α-SMA^+^/DAPI) cells in the 30 μm fibrous cap area of these mice (Figure 4H and Supplemental Figure 2, D and E). Interestingly, ABT-263 treatment increased EC (Cdh5-eYFP^+^/DAPI) in the fibrous cap and lesions but did not increase endothelial-derived α-SMA^+^ (Cdh5-eYFP^+^ α-SMA^+^/ α-SMA^+^) cells (Figure 4, I and J, and Supplemental Figure 2, F–H).

### ABT-263 increased apoptosis in nonsenescent EC.

**Figure 5:**
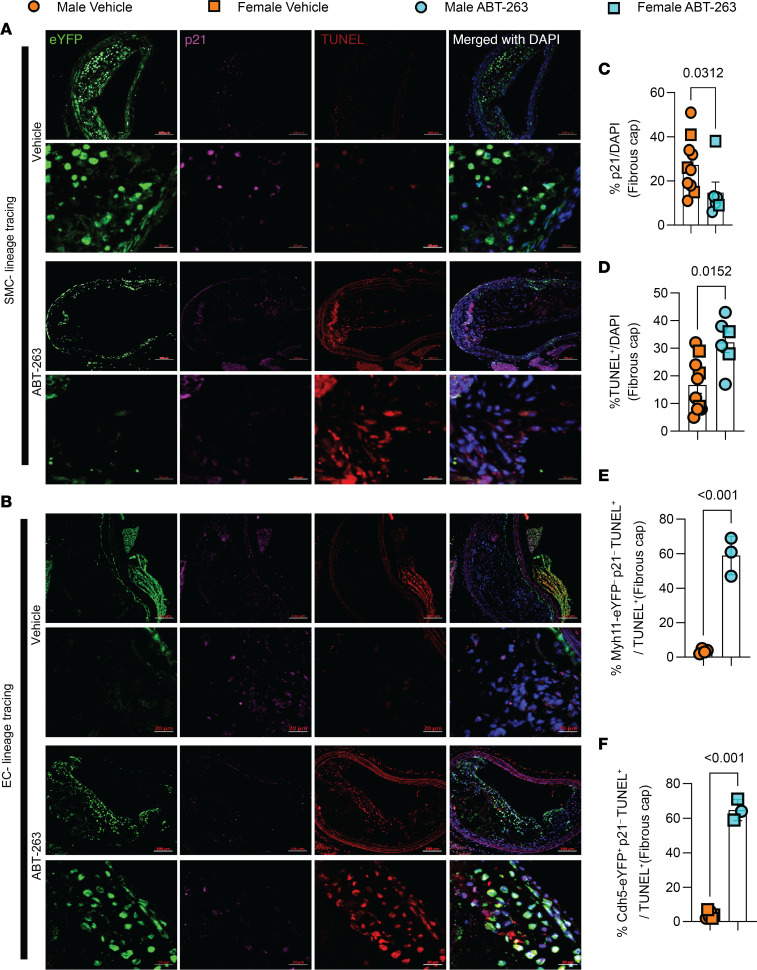
ABT-263 treatment (100mg/kg/bw) of SMC (Myh11-CreER^T2^-eYFP) and EC (Cdh5-CreER^T2^-eYFP) lineage tracing *Apoe^–/–^* mice with advanced atherosclerotic lesions was associated with a marked increase in nonsenescent endothelial cell apoptosis. (**A**) Representative confocal images of costaining for eYFP (for detecting SMC), p21 (detecting senescent cells), TUNEL (detecting apoptotic cells), and DAPI in advanced BCA lesions from SMC lineage tracing *Apoe^–/–^*mice were fed a WD for 18 weeks, followed by 100 mg/kg/bw ABT-263 treatment on WD for 6 weeks. The confocal images show a maximum intensity projection ×20 zoom. Scale bar: 100 μm and 20 μm (zoomed-in images). (**B**) Representative confocal images of costaining for eYFP (for detecting EC), α-SMA^+^, and DAPI in advanced BCA lesions from EC lineage tracing *Apoe^–/–^* mice were fed a WD for 18 weeks followed by 100 mg/kg/bw ABT-263 treatment on WD for 6 weeks. Scale bars: 100 μm (top); 20 μm (bottom)**.** (**C**) Quantification of the percentage of p21^+^ (p21^+^/DAPI^+^) cells in the fibrous cap from **A** and **B**. (**D**) Quantification of the percentage of TUNEL^+^ (TUNEL^+^/DAPI^+^) cells in the fibrous cap from **A** and **B**. (**E**) Non–SMC-derived nonsenescent apoptotic cells (Myh11-eYFP^–^ p21^–^ TUNEL^+^/TUNEL^+^) of all apoptotic cells from **A**. (**F**) EC-derived nonsenescent apoptotic cells (Cdh5-eYFP^+^ p21^–^ TUNEL^+^/TUNEL^+^) of all apoptotic cells from **B**. Mann-Whitney *U* tests were used for statistical analysis in **C**–**F**. Biologically independent animals are indicated as individual dots. Data are shown as mean ± SEM (**C** and **D**) and ± SD (**E** and **F**). The *P* values are indicated on the respective graphs.

To ascertain whether ABT-263 exclusively induces apoptosis in senescent cells or if it also affects other nonsenescent cells in the BCA, we conducted costaining on ABT-263–treated BCA lesions with lineage-traced SMC and EC, using p21 as a senescence marker and TUNEL as an apoptosis marker (Figure 5, A and B). Overall, ABT-263 reduced the presence of senescent (p21^+^/DAPI) cells and increased the occurrence of apoptotic (TUNEL^+^) cells within the fibrous cap (Figure 5, C and D). However, the increased apoptotic cells were neither derived from SMC nor senescent (Myh11-eYFP^–^ p21^–^ TUNEL^+^/TUNEL^+^) cells (Figure 5E); rather, they originated from EC (Cdh5-eYFP^+^ p21^–^ TUNEL^+^/TUNEL^+^), constituting nonsenescent apoptotic cells (Figure 5F). Taken together, results show that ABT-263 treatment at 100 mg/kg/bw had no effect on lesion size but markedly reduced the probability of survival. This treatment regimen was also associated with major detrimental changes in lesion composition, including reduced α-SMA^+^ fibrous cap thickness and SMC investment into the fibrous cap. In addition, ABT-263 treatment prevented adaptive increases in investment of EC-derived cells into the fibrous cap via EndoMT to myofibroblast transitions that we have shown markedly increases when SMC investment into the fibrous cap of lesions is impaired (9).

### ABT-263 dose-dependently reduced SMC and EC viability.

**Figure 6:**
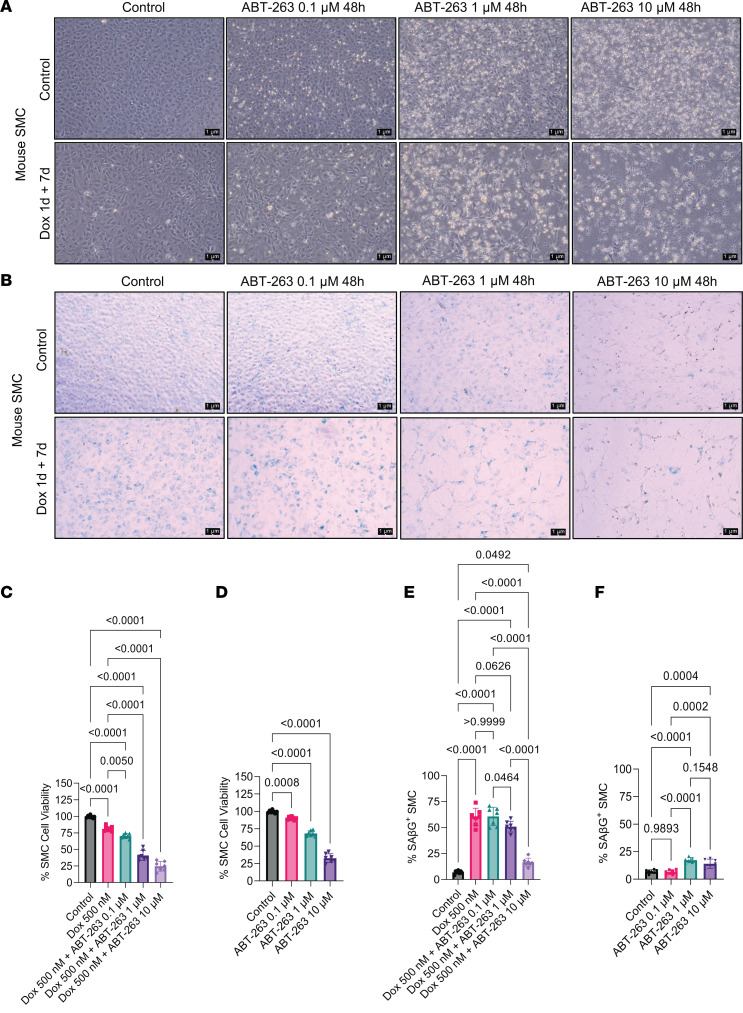
ABT-263 dose-dependently reduced smooth muscle cell viability. (**A**) Mouse smooth muscle cells were treated with Dox for 1 day+ 7 days of recovery; they were then treated with different concentrations (0.1 μM, 1 μM, and 10 μM) of ABT-263. Representative photographs at the end of the treatment were taken with Leica of 10× zoom. Scale bar: 1 μm. (**B**) At the end of the treatment, we aspirated the media and performed the SAβG assay as described in Methods. Images were taken in 10× zoom of Leica microscope. Scale bar: 1 μm. (**C** and **D**) Percentage of SMC cell viability was measured with trypan blue assay with and without Dox and ABT-263. (**E** and **F**) Percentage of SMC senescence measured from **B**. The 1-way ANOVA method was used for statistical analyses for **C**–**F**. Each well of the plate indicated as individual dots. Data are shown as mean ± SEM. The *P* values are indicated on the respective graphs. All the cell culture experiments in this figure repeated 3 times.

To determine whether ABT-263 induces cell death exclusively in senescent cells, we treated mouse SMC with doxorubicin (Dox) for 1 day to induce cell senescence, followed by a 7-day recovery in normal media. Subsequently, the cells were treated with varying doses of ABT-263 for 48 hours, and photomicrographs were captured after the treatment (Figure 6A). Cell viability was assessed using the trypan blue assay. Interestingly, ABT-263 dose-dependently reduced cell viability not only in the Dox-treated group but also in the control group of proliferating cells (Figure 6, C and D). Additionally, at the end of the treatments, media were aspirated, and the plates were washed with PBS. A SAβG assay was performed (Figure 6B). At 0.1 μM concentration, ABT-263 did not reduce Dox-induced senescence but reduced cell viability of non–Dox-treated cells (Figure 6, D–F). However, ABT-263 reduced Dox-induced senescence by 13% at a concentration of 1 μM compared with the Dox-treatment group (Figure 6, E and F). It also induced cell death by 40% in SMC without Dox treatment (Figure 6, E and F). A concentration of 10 μM ABT-263 exhibited high SMC toxicity, including increasing cell death by ~75% (Figure 6, A, B, E, and F). In summary, ABT-263 treatment induced cell death in nonsenescent cultured mouse aortic SMC.

**Figure 7:**
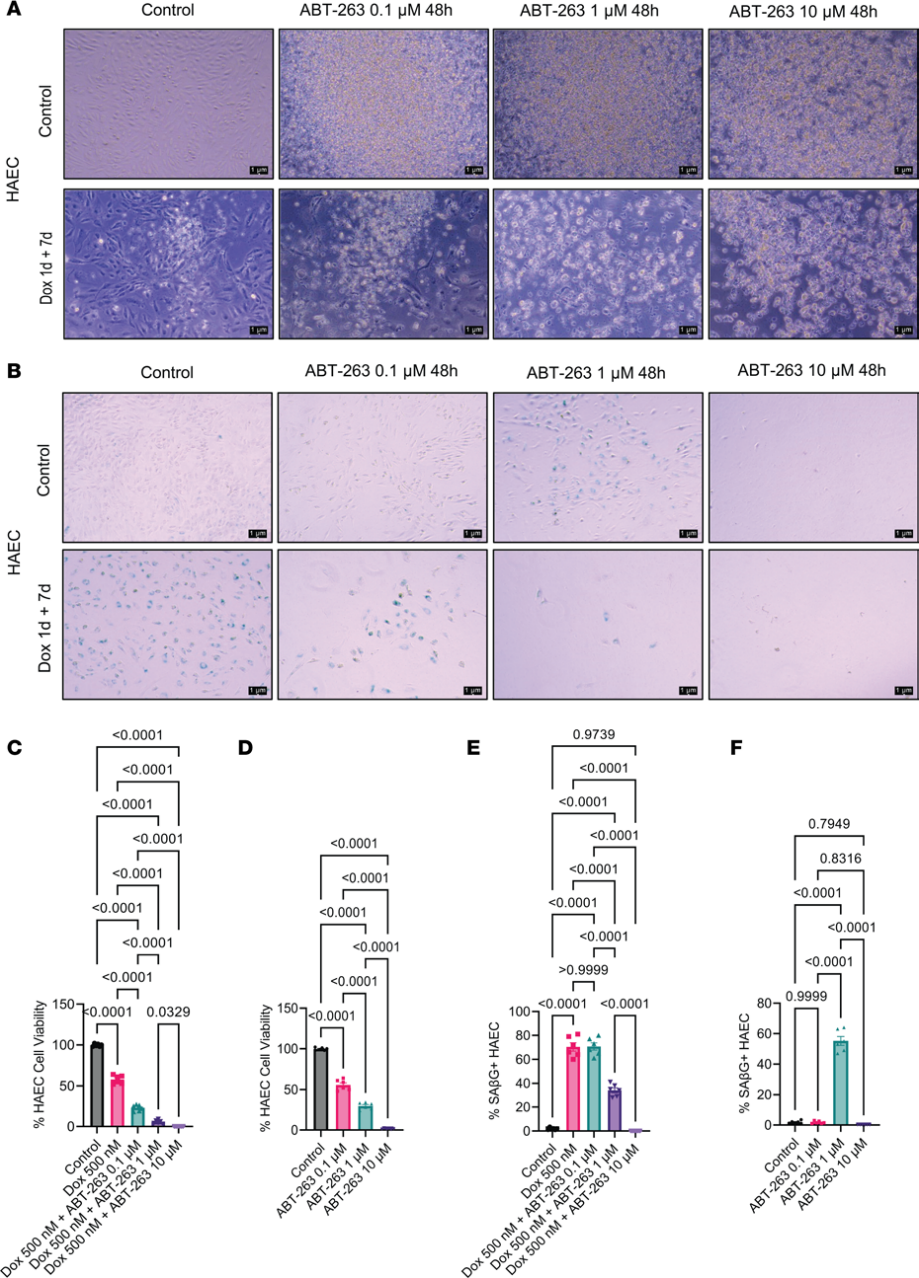
ABT-263 dose-dependently reduced endothelial cell viability. (**A**) HAEC obtained from ATCC were treated with Dox for 1 day+ 7 days of recovery; they were then treated with different concentrations (0.1 μM, 1 μM, and 10 μM) of ABT-263, and representative photographs at the end of the treatment were taken with Leica of 10× zoom. Scale bar: 1 μm. (**B**) At the end of the treatment, we aspirated the media and performed the SAβG assay as described in Methods, and images were taken in 10× zoom of Leica microscope. Scale bar: 1 μm. (**C** and **D**) Percentage of SMC cell viability was measured with trypan blue assay with and without Dox and ABT-263. (**E** and **F**) Percentage of HAEC senescence measured from **B**. The 1-way ANOVA method was used for statistical analyses in **C**–**F**. Each well of the plate indicated as individual dots. Data are shown as mean ± SEM. The *P* values are indicated on the respective graphs. All the cell culture experiments in this figure were repeated 3 times.

To ascertain whether ABT-263 induced cell death in senescent human aortic EC (HAECs), we replicated the experiment shown in Figure 6 but using HAECs. The HAECs were treated with and without Dox, as well as with and without 3 different doses of ABT-263. At the end of the treatment, the photographs revealed ABT-263–induced cell death (floating cells) even at a concentration of 0.1 μM (Figure 7A). A dose-dependent reduction in cell viability was observed with ABT-263 (Figure 7, A–D). At a concentration of 10 μM ABT-263, there were nearly no viable cells (Figure 7B), regardless of the presence or absence of Dox. Furthermore, the SAβG assay indicated a reduction in Dox-induced cell senescence, but it also led to the death of nonsenescent cells (Figure 7B). Unexpectedly, a concentration of 0.1 μM ABT-263 did not decrease the percentage of senescent cells, yet it reduced cell viability by 50%. Moreover, 1 μM ABT-263 reduced senescent cells while causing a 99% reduction in cell viability (Figure 7, C–F). Finally, 10 μM ABT-263 decreased senescent cells but did not leave any viable cells (Figure 7, C–F). These observations, and results from the previous sections, strongly suggest that ABT-263 might be killing nonsenescent SMC and EC, which are crucial for maintaining the fibrous cap of atherosclerotic plaques.

### Apoe^–/–^ mice with advanced lesions treated with a reduced dose of ABT-263 also had increased mortality and detrimental changes in lesion composition.

**Figure 8:**
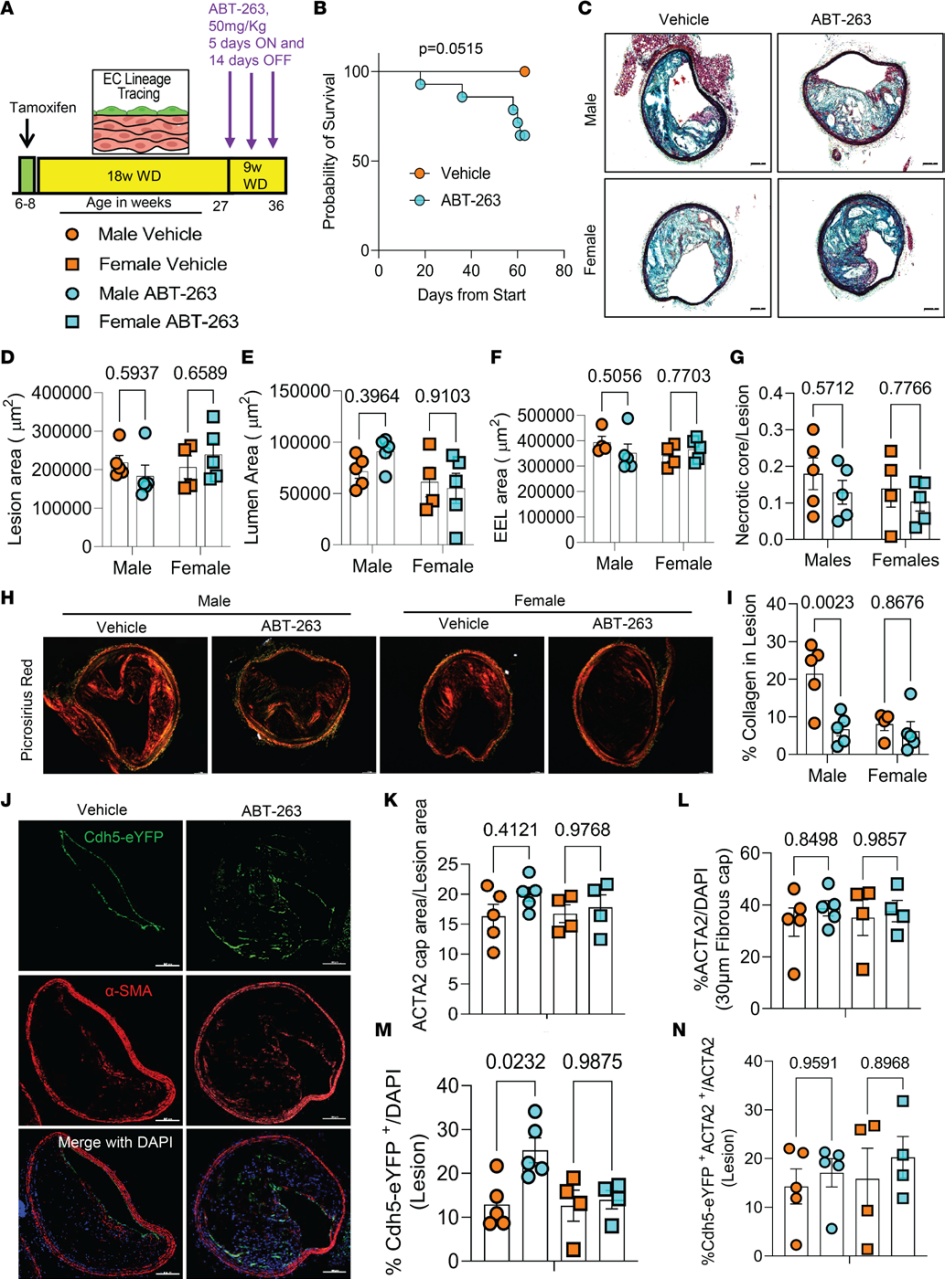
Treatment of *Apoe^–/–^* mice with advanced lesions with a reduced dose (50mg/kg/bw) of ABT-263 decreased collagen content within BCA lesions and was also associated with increased mortality. (**A**) Experimental design. EC lineage tracing *Apoe^–/–^* mice were fed a WD for 18 weeks followed by 50 mg/kg/bw ABT-263 treatment on WD for 9 weeks. (**B**) Probability of survival (Kaplan-Meier curve). (**C**) Representative 10× images of MOVAT staining of the BCA. Scale bar: 100 μm. (**D**) Lesion area from **C**. (**E**) Lumen area from **C**. (**F**) External elastic lamina (EEL) area from **C**, for outward remodeling. (**G**) Necrotic core area normalized to lesion size. (**H**) Representative 10× images of Picrosirius red staining on BCA. Scale bar: 100 μm. (**I**) Quantification of mature (red) collagen content normalized to lesion area from **H**. (**J**) Representative confocal images of costaining for eYFP (for detecting EC), α-SMA^+^, and DAPI in advanced BCA lesions. Scale bars: 100 μm. (**K**) α-SMA^+^ cap area normalized to lesion area (α-SMA^+^ cap area/lesion area). (**L**) Quantification of the percentage α-SMA^+^ (α-SMA^+^/DAPI) cells in the fibrous cap. (**M**) Quantification of the percentage EC-derived (Cdh5-eYFP^+^/DAPI^+^) cells in the lesion. (**N**) Quantification of the percentage EC-derived α-SMA^+^ (Cdh5-eYFP^+^ α-SMA^+^/α-SMA^+^) cells in lesions. The 2-way ANOVA method was used for statistical analysis in **D**, **G**, and **K**–**L**, and biologically independent animals are indicated as individual dots. Data are shown as mean ± SEM. A Mantel-Cox test was used for statistical analysis in **B**. The *P* values are indicated on the respective graphs.

Given the multiple detrimental effects of treating WD-fed *Apoe^–/–^* mice with ABT-263 at 100 mg/kg/bw, we repeated the preceding studies with 50 mg/kg/bw of ABT-263. EC lineage tracing *Apoe^–/–^* mice were fed a WD for 18 weeks followed by treatment with ABT-263 at half our original dose (i.e., 3 cycles of 50 mg/kg/bw) (Figure 8A). Contrary to our expectations, even this lower dose of ABT-263 was associated with increased mortality (Figure 8B). Similar to the higher dose of ABT-263, there were no changes in lesion size, lumen area, outward remodeling, or necrotic core area of BCA lesions (Figure 8, B–G). There were also no reductions in aortic root lesion area (Supplemental Figure 3, A–C) in male or female mice. Surprisingly, the lower dose of ABT-263 reduced collagen content in male *Apoe^–/–^* mice (Figure 8, H and I). Unlike the 100 mg/kg dose, treatment at the lower dose did not reduce α-SMA^+^ cap thickness, area, or α-SMA^+^ cells in the fibrous cap or lesions (Figure 8, J–L, and Supplemental Figure 4, A and B). As was true in the preceding higher ABT-263 dosage studies, the lower-dose study of ABT-263 was associated with increased EndoMT (Cdh5-eYFP^+^/DAPI) in male *Apoe^–/–^* mice, but there was no change in endothelial-derived α-SMA^+^ (Cdh5-eYFP^+^ α-SMA^+^/ α-SMA) cells in the lesions or 30 μm fibrous cap area (Figure 8, M and N, and Supplemental Figure 4, C and D). Taken together, even the reduced dose of ABT-263 increased mortality and did not show beneficial changes in plaque size and composition.

### Low-dose ABT-263 treatment of WD-fed Apoe^–/–^ mice reduced plasma Cxcl5 and was associated with fibrous liver phenotype.

To determine if ABT-263 treatment reduces SASPs and proinflammatory cytokine levels, we performed luminex assays on plasma samples. Here we discovered that ABT-263 did not reduce SASPs or cytokines, such as *IL-1β*, *IL-1*α, *IL-6*, *Mcp-1*, *Tnf-*α, and *Ifn-*γ (Supplemental Figure 5). However, ABT-263 treatment reduced *LIX* (*Cxcl5*) levels in female *Apoe^–/–^* mice (Supplemental Figure 5). *Cxcl5* has an atheroprotective role, in that inhibition of *Cxcl5* has previously been shown to induce significant macrophage foam cell accumulation in murine atherosclerotic plaques (36).

Mice treated with ABT-263 also showed an increase in Masson trichrome–positive fibrous tissue or compromised normal liver phenotype as compared with vehicle treatment (Supplemental Figure 6). However, ABT-263 treatment did not result in changes in alanine aminotransferase (ALT) and aspartate aminotransferase (AST) levels in the plasma (Supplemental Figure 6, F and G), suggesting that the ABT-263–induced increase in liver damage and fibrosis may be associated with other factors or mechanisms. Although ABT-263 did not reduce lesion size, it reduced cholesterol and LDL levels in male but not female *Apoe^–/–^* mice (Supplemental Figure 7, B and C). Triglyceride levels were not changed with ABT-263 treatment (Supplemental Figure 7D). ABT-263 treatment did not increase thrombocytopenia (Supplemental Figure 7E). Moreover, ABT-263 did not result in changes in basophils, lymphocytes, WBC, RBC, nucleated RBC, hemoglobin, monocytes, or neutrophils in the blood (Supplemental Figure 8). Also, ABT-263 treatment did not change the body weights (Supplemental Figure 9).

## Discussion

Senescent cells contribute to age-associated diseases (37), and recent murine studies have tested the ability of senolytic agents to remove these senescent cells and to potentially treat a number of major diseases, including atherosclerosis (25, 26, 29, 30), neurodegeneration (38), pulmonary fibrosis (39), diabetic chronic kidney disease (40), and cancer (28). Indeed, multiple ongoing clinical trials with senolytic drugs are in progress targeting various diseases, including Alzheimer’s (NCT0463124) and diabetic chronic kidney disease (NCT02848131) (41). These clinical trials are based on the belief that selective removal of these senescent cells will be beneficial. However, the results of the present study show that one of the most popular senolytic drugs, ABT-263, has multiple detrimental effects on *Apoe^–/–^* mice with advanced atherosclerosis; these effects include causing a reduced probability of survival by 50%–60% that may be due to a reduced α-SMA^+^ fibrous cap thickness and a 90% reduction in SMC within the lesions. Indeed, our results suggest that the majority of SMC-derived cells within lesions, including those that are critical for the formation and maintenance of the protective fibrous cap, are particularly sensitive to ABT-263–induced clearance. Consistent with this possibility, Leeper and coworkers demonstrated that clonal expansion of lesion SMC is dependent on those SMC escaping efferocytosis at least in part by activating the antiphagocytic molecule CD47 (42). Results indicate that ABT-263 may be inducing apoptosis of nonsenescent SMC and that, with long-term use, it may increase the risk for the development of unstable advanced atherosclerotic lesions and the incidence of myocardial infarction (MI) or stroke. This may be particularly severe, since our data also show that ABT-263 prevented adaptive increases in investment of EC-derived cells into the fibrous cap via beneficial EndoMT to myofibroblast transitions that we have shown normally occur when SMC investment into fibrous cap of lesions is impaired (9).

At first glance, our results showing multiple detrimental changes in lesion pathology and a marked increase in mortality appear to be at odds with previous studies in the field reporting beneficial effects of ABT-263 treatment of atherosclerotic mice (25, 26). The reasons for these differences are unclear but likely include the following key variables between studies. First, to better match clinical paradigms of treating elderly patients with advanced disease, we did a late-stage intervention study (35) involving initiation of ABT-263 treatment in *Apoe^–/–^* mice after 18 weeks of WD (TD.88137; 42% calories from fat) feeding so that they already have highly advanced BCA lesions closely resembling advanced human lesions both morphologically (2) and in cellular composition of lesions, as determined by scRNA-Seq analysis (15). In contrast, previous studies showing beneficial effects of ABT-263 were either prevention studies, where treatment was initiated in very young mice at the beginning of WD feeding (25), or intervention studies following just 12–14 weeks of WD feeding of *Ldlr^–/–^* mice, at which time lesions are still in the early stage (26). Second, to model patients with undiagnosed advanced disease or with poor lipid management, we elected to continue mice on a WD during the 9-week treatment period (i.e., 27 weeks of WD with or without ABT-263 treatment during the last 9 weeks). In contrast, to mimic the clinically relevant context of patients with highly effective medical management of proatherogenic lipids, Childs et al. (26) fed a WD to *Ldlr^–/–^* mice for 12 weeks, followed by 9 weeks of LFD with or without ABT-263. They observed multiple beneficial effects of ABT-263 treatment, including thickening of the fibrous cap of aortic lesions. Given that we modeled our intermittent ABT-263 dosing regimen to that of Childs et al. (25), it is likely that the high mortality observed in our studies with ABT-263 is due, at least in part, to prolonged hyperlipidemia (Supplemental Figure 10). However, it remains to be determined whether our ABT-263–induced increase in mortality is due to thromboembolic events secondary to plaque destabilization, increased liver toxicity, and/or other mechanisms. Nevertheless, no matter what the cause of death with ABT-263, it is concerning, given the extensive clinical (43, 44) testing in progress. Indeed, results suggest that it may be prudent to exclude patients with poorly controlled lipids, systemic inflammation, and/or advanced atherosclerosis from those studies.

Previous studies reported that EC-like (24) and SMC-like (20) cells undergo senescence that is associated with telomere shortening in human atherosclerotic lesions. The results of the present study show that KO of *Klf4* in SMC was associated with reduced expression of prosenescence markers but preserved expression of the antisenescence marker *Tert^+^*. It is not clear how the loss of *Klf4* in SMC preserved *Tert^+^* expression. However, it is probably the result of loss of *Klf4-*induced repression of *Tert*, given previous studies showing that *Klf4* downregulates *hTERT* expression and telomerase activity to inhibit lung carcinoma growth (45). Also, the KD of *Klf4* in HUVECs in hyperglycemic conditions increased *hTERT* expression (46). Telomerase maintains the telomere length, and the telomerase deficiency (*Trf1*- and *Tert*-deficient mice) in mice leads to aplastic anemia. *Tert* gene therapy improved telomere length and blood count in these *Tert*-deficient mice (47). Furthermore, recent *Tert* gene therapy in the mice reduced vascular senescence and increased lifespan (48), improved myocardial revascularization and tissue repair (49, 50), and reduced neurodegeneration associated with short telomeres (51). Given the multiple beneficial effects of *Tert* therapy in the preclinical mouse models, future studies need to evaluate if the *Tert* therapy would show beneficial effects on advanced atherosclerosis.

Given the strong link between aging and atherosclerosis, a key question is to what extent any of the current mouse models commonly used adequately address the role of cell senescence and aging in late-stage lesion pathogenesis, including plaque rupture or erosion leading to myocardial infarction or stroke. Indeed, for reasons having to do with minimizing the cost and time of experiments rather than scientific rigor, most studies of atherosclerosis in mice are limited to studying early-stage fatty streak formation and aortic root lesion area in *Apoe^–/–^* or *LDLr^–/–^* mice following WD feeding of just-weaned mice for 10–12 weeks. Studies of fatty streak provide little if any insight regarding late-stage lesion pathogenesis and humans do not develop aortic root lesions. Thus, even correcting for lifespan differences between mice and humans, the mice used in these studies cannot be considered to be aged, although their lesions do contain numerous cells expressing putative markers of cell senescence. The studies described herein focused on treating much older mice with advanced BCA lesions that closely resemble human lesions based on morphology (2) and scRNA-Seq analyses (15). We contend that they provide a better model for elucidating the relationship between aging, senescence, and late-stage atherosclerotic lesion pathogenesis. However, there is a need for further studies in mouse models that, unlike *Apoe^–/–^* and *Ldlr^–/–^* mice, develop coronary lesions that undergo spontaneous plaque rupture and MI in mice that are truly aged (i.e., > 1.5 years) and in mice that have modest increases in plasma cholesterol and lipid levels similar to high CAD risk patients.

In conclusion, ABT-263 treatment of WD-fed *Apoe^–/–^* mice with advanced atherosclerosis and persistent hyperlipidemia had multiple unexpected detrimental effects, including the induction of reduced Myh11-eYFP^+^ SMC–derived cells within lesions and the fibrous cap and decreased α-SMA^+^ fibrous cap thickness. ABT-263 treatment also prevented adaptive increases in investment of EC-derived cells into the fibrous cap via beneficial EndoMT-to-myofibroblast transitions and increased mortality. Removing the senescent cells from the plaque is conceptually a good idea, but given the multiple detrimental effects of ABT-263 in our WD-fed *Apoe^–/–^* mice, further preclinical studies with senolytic drugs are needed to identify factors and mechanisms that modulate senolytic drugs biological effects before planning a large clinical trial targeting atherosclerosis.

## Methods

### Mice

The Myh11-CreER^T2^-eYFP and Cdh5-CreER^T2^-eYFP *Apoe^–/–^* mice used in the intervention study have been described in our previous studies (4, 9). In addition, Myh11-CreER^T2^-eYFP *Klf4* WT versus KO *Apoe^–/–^* mice were used for the studies related to loss of *Klf4* in SMC experiments as previously reported (4).

### Diet and treatment

#### ABT-263 intervention studies on advanced atherosclerotic mice.

In Myh11-CreER^T2^-eYFP and Cdh5-CreER^T2^-eYFP *Apoe^–/–^* mice, Cre recombinase was activated with a series of 10 tamoxifen injections (1 mg/day/mouse; Sigma-Aldrich, T-5648) over a 2-week period. One week after the tamoxifen treatment, mice were switched from a normal chow diet (Harlan Teklad, TD.7012) to a high fat WD, containing 21% milk fat and 0.15% cholesterol (Harlan Teklad, TD.88137) for 18 weeks, followed by 100 mg/kg/bw ABT-263 treatment for 6 weeks (2 cycles of 5 days on and 14 days off) or 50 mg/kg/bw ABT-263 treatment for 9 weeks (3 cycles of 5 days on and 14 days off). ABT-263 (S1001) was obtained from Selleck, formulated in vehicle (PBS [Thermo Fisher Scientific, catalog 10010031] with 15% dimethylsulfoxide [MilliporeSigma, catalog 5.89569] and 7% Tween-20 [Sigma Aldrich catalog P1379]) and injected i.p. at a dose of 100 mg/kg/bw or 50 mg/kg/bw as shown in the figures, figure legends, and Results.

### Atherosclerotic plaque morphometry

Paraformaldehyde-fixed paraffin-embedded BCAs were serially cut into 10 μm–thick sections from the aortic arch to the bifurcation of the right subclavian artery. For morphometric analysis, we performed modified Russell-Movat staining on 3 locations along the BCA at 150 μm, 450 μm, and 750 μm, from the aortic arch as previously described (9). The lesion, lumen, external elastic lamina (outward remodeling), and necrotic core areas, as well as the internal elastic lamina area, were measured on digitized images of the Movat staining using Fiji (ImageJ) software. Picrosirius red staining was performed to assess collagen content, and digitized images of the Picrosirius red staining was measured using Fiji software. Masson trichrome staining was performed to assess the fibrous tissue in the liver sections.

### Immunofluorescence staining

BCA sections were deparaffinized and rehydrated in xylene and ethanol series. After antigen retrieval (H-3300-250, Vector Laboratories), sections were blocked with fish skin gelatin–PBS (6 g/L) containing 10% horse serum for 1 hour at room temperature. Slides were incubated with the following antibodies: mouse monoclonal α-SMA–Cy3 (4.4 μg/mL, clone 1A4, C6198, Sigma-Aldrich), goat polyclonal anti-GFP (4 μg/mL, ab6673, Abcam) for detection of eYFP, and rabbit polyclonal p21 (18 μg/mL, 10355-1-AP, Proteintech). TUNEL (30074, Bioutium) was used for staining. The secondary antibodies donkey anti-goat AF-488 (Invitrogen, A11055) conjugated to Alexa 488 (5 μg/mL) and donkey anti-rabbit conjugated to Alexa 647 (A31573, 5 μg/mL) were used (Thermo Fisher Scientific), and DAPI (0.05 mg/mL, D3571) was used as a nuclear counterstain; slides were mounted using Prolong Gold Antifade (P36930), purchased from Thermo Fisher Scientific.

### Imaging

Movat and Picrosirius red staining of BCAs and Masson trichrome staining of liver sections were imaged by using a Leica thunder imager microscope. Image acquisition was performed with Leica software. Digitized images were analyzed with Fiji software. Immunofluorescence staining was imaged using a Zeiss LSM880 airy scan confocal microscope to acquire a series of *Z* stack images at 1 μm intervals. Zen 2009 Light Edition Software (Zeiss) was used for the analysis of each *Z* stack image, and single-cell counting was performed for phenotyping and quantifying the cell population comprised within the 30 μm–thick layer proximal to the lumen (i.e., fibrous cap area). Assessment of α-SMA^+^ cap thickness (normalized to lesion) was performed using Zen 2009 Light Edition Software. Maximal intensity projection of representative images was used to generate the representative images included in the figures.

### Western blotting

The method has been described previously (52). The primary antibodies used for p16 were from Abcam (2 μg/mL, ab189034), and Klf4 (1 μg/mL, CST4038T) and β-actin (1 μg/mL, 4967S) were from Cell Signaling Technology. The anti–rabbit IgG (A4914) HRP-conjugated secondary antibody was purchased from Sigma-Aldrich.

### SAβG staining

SAβG staining of aortas for senescent cells was examined using a senescence detection assay kit (MilliporeSigma, KAA002) (52). Freshly isolated aortas were kept on ice in a 12-well plate containing PBS until all the aortas were harvested. Aortas were washed twice with the PBS and then fixed with the diluted fixation solution provided in the kit for 10 minutes, as per the manufacturer’s instructions. After the fixation, aortas were washed 3 times with PBS, and then 1 mL of freshly prepared SAβG solution was added to a well of 12-well plates containing 1 aorta per well. Then, plates were wrapped with aluminum foil to avoid light exposure and placed in a 37°C incubator for 24 hours. After that, aortas were washed 3 times with PBS, and photographs were taken with a mobile phone camera. Similar to aorta staining, at the end of the treatment, cell culture plates containing cells were fixed and stained with SAβG stain and imaged with Leica microscope.

### Statistics

Mann-Whitney U-tests were used for statistical analyses when there were two groups. The 1-way ANOVA method was used for statistical analyses comparing multiple groups. A repeated measure 2-way ANOVA method was used for statistical analysis comparing the two factors are investigated simultaneously to measure the interaction of the two factors influencing the values of a variable. A Mantel-Cox test was used for Kaplan-Meier curve. Statistics were performed using GraphPad prism 9. Data are presented as mean ± SEM. Animal numbers and type of statistical analysis done are reflected within figures and figure legends. *P* ≤ 0.05 was considered statistically significant.

### Study approval

The University of Virginia IACUC approved animal protocols (protocol 2400).

### Data availability

Values for all data points in graphs are reported in the Supporting Data Values file.

## Author contributions

SK conceptualized, designed, and performed the bulk of the experiments, validation, data collection, and analysis and interpretation of data, as well as contributed significantly to development of methodology, manuscript writing, and editing. VK contributed to data collection; analysis of indices of stability; Movat, Picrosirius analysis, and IFC staining; confocal imaging; counting the single cells of confocal images; harvesting mice; collecting samples; data interpretation; and manuscript editing. LSS contributed to data analysis, data interpretation, and manuscript editing. GKO supervised the entire project, conceptualized experiments, and contributed to data interpretation, funding, manuscript writing, and editing.

## Supplementary Material

Supplemental data

Supporting data values

## Version 1 01/23/2024

Electronic publication
