# Supplementary material for: Treatment of advanced atherosclerotic mice with ABT-263 reduced indices of plaque stability and increased mortality
Source: JCI Insight. 2024 Jan 23;9(2):e173863. doi: 10.1172/jci.insight.173863 (PMC10906456; doi:10.1172/jci.insight.173863)
Supplement: Supplemental data [file jciinsight-9-173863-s206.pdf]

Treatment of advanced atherosclerotic mice with ABT-263 reduced indices of plaque stability and increased mortality

Santosh Karnewar, Vaishnavi Karnewar, Laura S. Shankman, Gary K. Owens*

Robert M. Berne Cardiovascular Research Center, University of Virginia-School of Medicine, 415 Lane Road, Suite 1010, Charlottesville, VA, 22908, USA.

Short Title: ABT-263 caused increased mortality in *Apoe*$^{-/-}$ mice.

Corresponding author:

Dr. Gary K. Owens

Univ. of Virginia School of Medicine

Robert M. Berne Cardiovascular Research Center

PO Box 801394

MR5 Building

Charlottesville, Virginia 22908-1394

Phone: 434-924-5993

Email: gko@virginia.edu

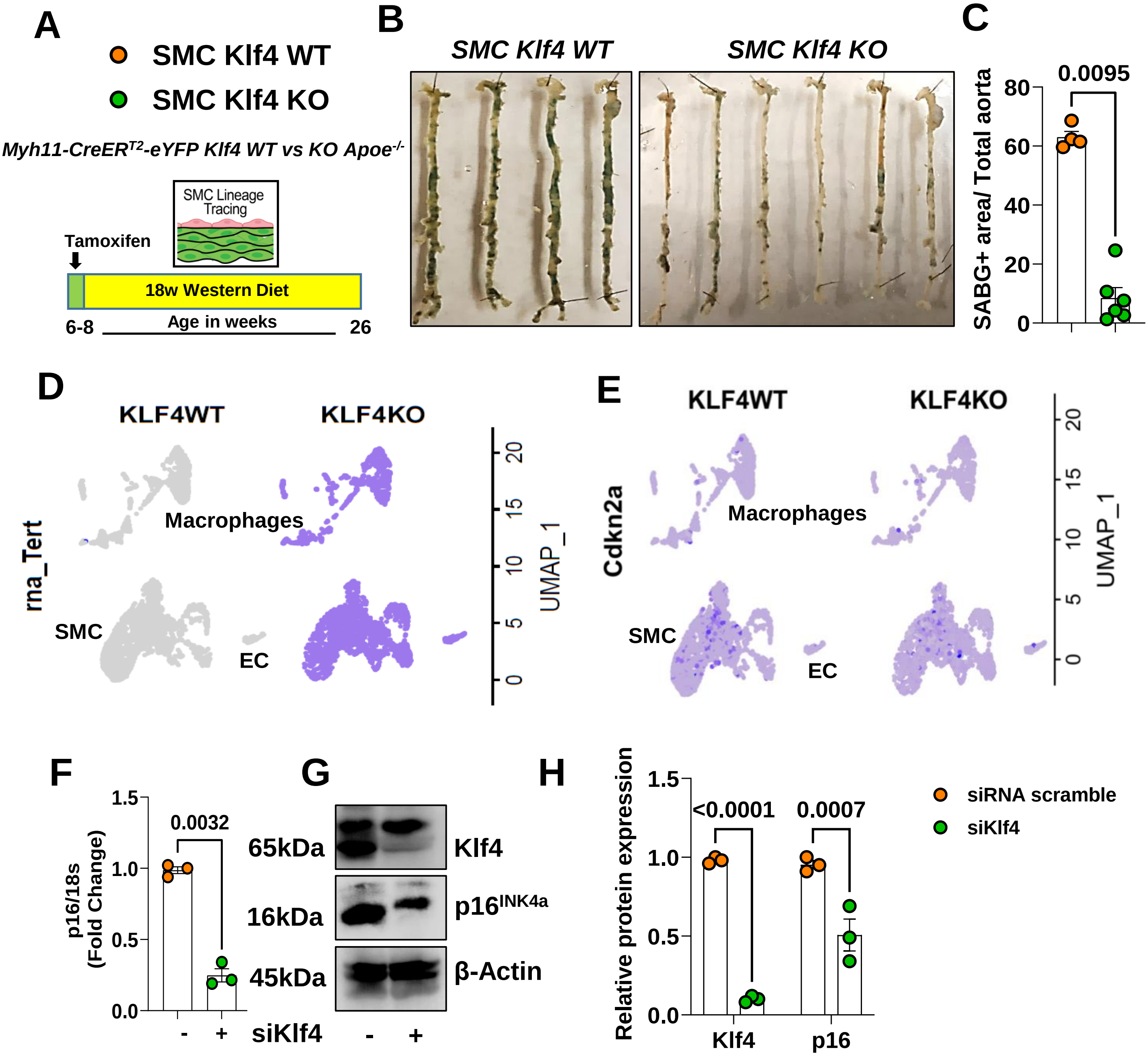

Supplemental Figure 1. SMC Klf4 KO reduces overall lesion senescence. (A) Experimental design, SMC lineage tracing Klf4 WT and KO Apoe-/- mice were injected with tamoxifen at 6 to 8 weeks of age and subsequently placed on a western diet (WD) for 18 weeks to induce advanced atherosclerosis. Freshly isolated aortas were then (B) SAβG stained, and (C) SAβG+ area normalized to the total luminal area of aortas shown in figure B. SAβG stained aortas were quantified using Fiji (ImageJ 1.53c) software on digitized images. UMAPs of (D) Tert and (E) p16 (Cdkn2a) from SMC Klf4 WT and KO scRNAseq data sets of micro-dissected BCA lesions. The blue dots indicate the presence of SMC-derived eYFP+ cells. Passage number 15 cultured murine aortic SMC were transfected with siKlf4 for 24h and (F) p16 mRNA levels were measured by qRT-PCR. (G) p16 protein levels were measured by western blot analyses. A Klf4 western blot was done to assess the efficiency of siRNA protein knock-down. β-Actin served as a control for quantification. (H) Relative protein expression of p16 and Klf4 was quantified with Fiji. Mann-Whitney U-tests were used for C, F and a two-way ANOVA was used for H. The error bars show the standard error of the mean (SEM). The p-values are indicated on the respective graphs.

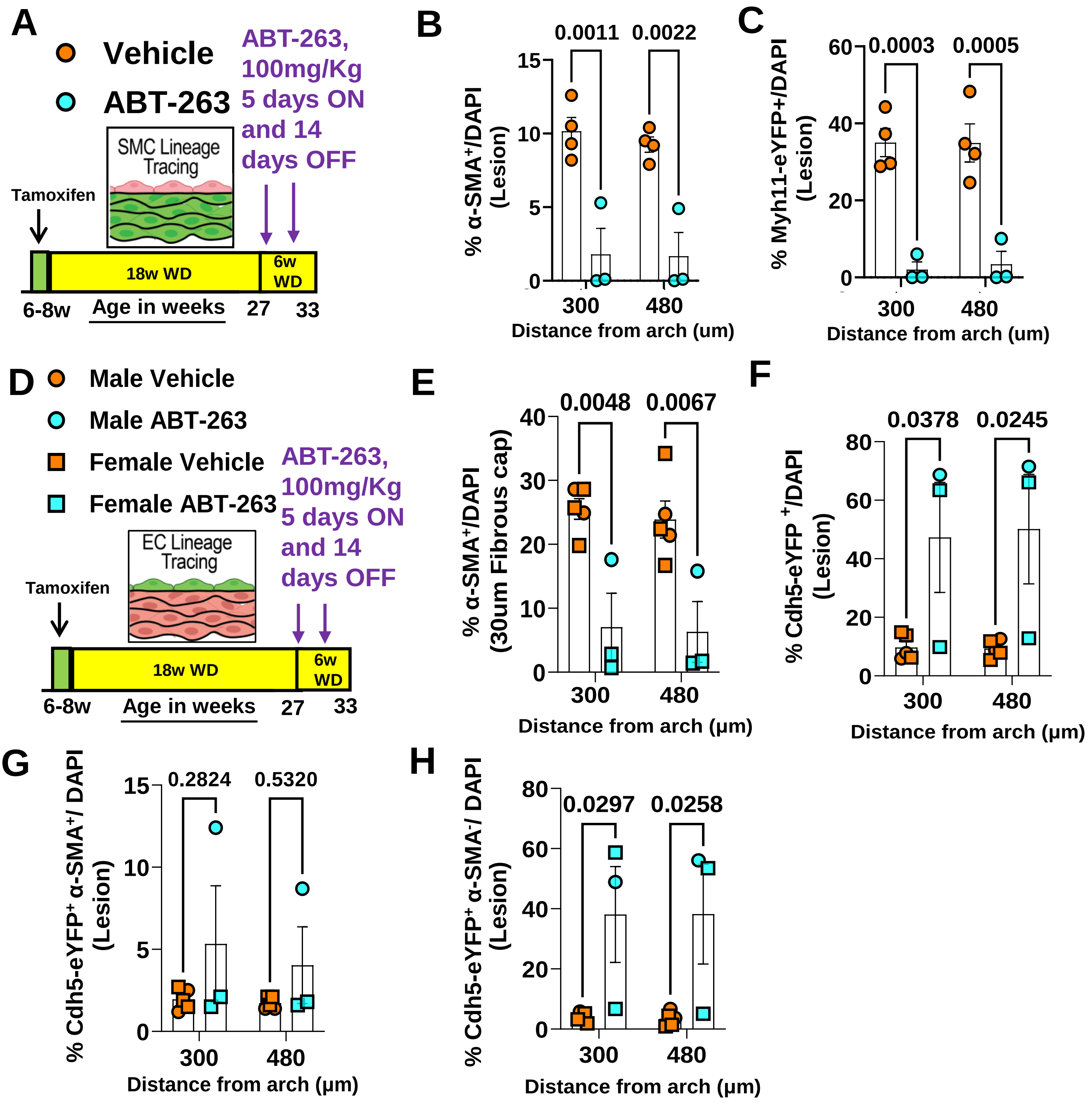

Supplemental Figure 2. Treatment of SMC (Myh11-CreER^{T2}-eYFP) and EC (Cdh5-CreER^{T2}-eYFP)-lineage tracing *Apoe$^{-/-}$* mice with advanced atherosclerotic lesions with the senolytic agent ABT-263 (100mg/kg/bw) decreased α-SMA^{+} cells in BCA lesions and the fibrous cap but increased EC derived cells in BCA lesions. (A) Experimental design for B and C: SMC-lineage tracing *Apoe$^{-/-}$* mice were fed a WD for 18 weeks followed by ABT-263 treatment on a Western diet (WD) for 6 weeks. **(B)** α-SMA^{+} cells of all DAPI^{+} cells and **(C)** Myh11-eYFP^{+} (SMC) of all DAPI^{+} cells in the lesion. **(D)** Experimental design for figures E-H: EC-lineage tracing *Apoe$^{-/-}$* mice were fed a WD for 18 weeks followed by 100mg/kg/bw ABT-263 treatment on WD for 6 weeks. Note: Males and females data combined due to low n-number (Circles indicate males, and Squares indicate females). **(E)** Percentage of α-SMA^{+} cells in the fibrous cap of all (DAPI^{+}) cells. **(F)** Percentage of endothelial cells (Cdh5-eYFP^{+}) of all (DAPI^{+}) cells in the lesions. **(G)** Percentage of EC-derived α-SMA^{+} (Cdh5-eYFP^{+} α-SMA^{+}) cells of all (DAPI^{+}) cells in the lesions. **(H)** Percentage of EC-derived but α-SMA negative (Cdh5-eYFP^{+} α-SMA^{-}) cells of all (DAPI^{+}) cells in the lesions. A two-way ANOVA was used for statistical analysis of panels of B-C and E-I. The p-values are indicated on the respective graphs. The error bars show the standard error of the mean (SEM). Independent animals are indicated as individual dots on the graphs.

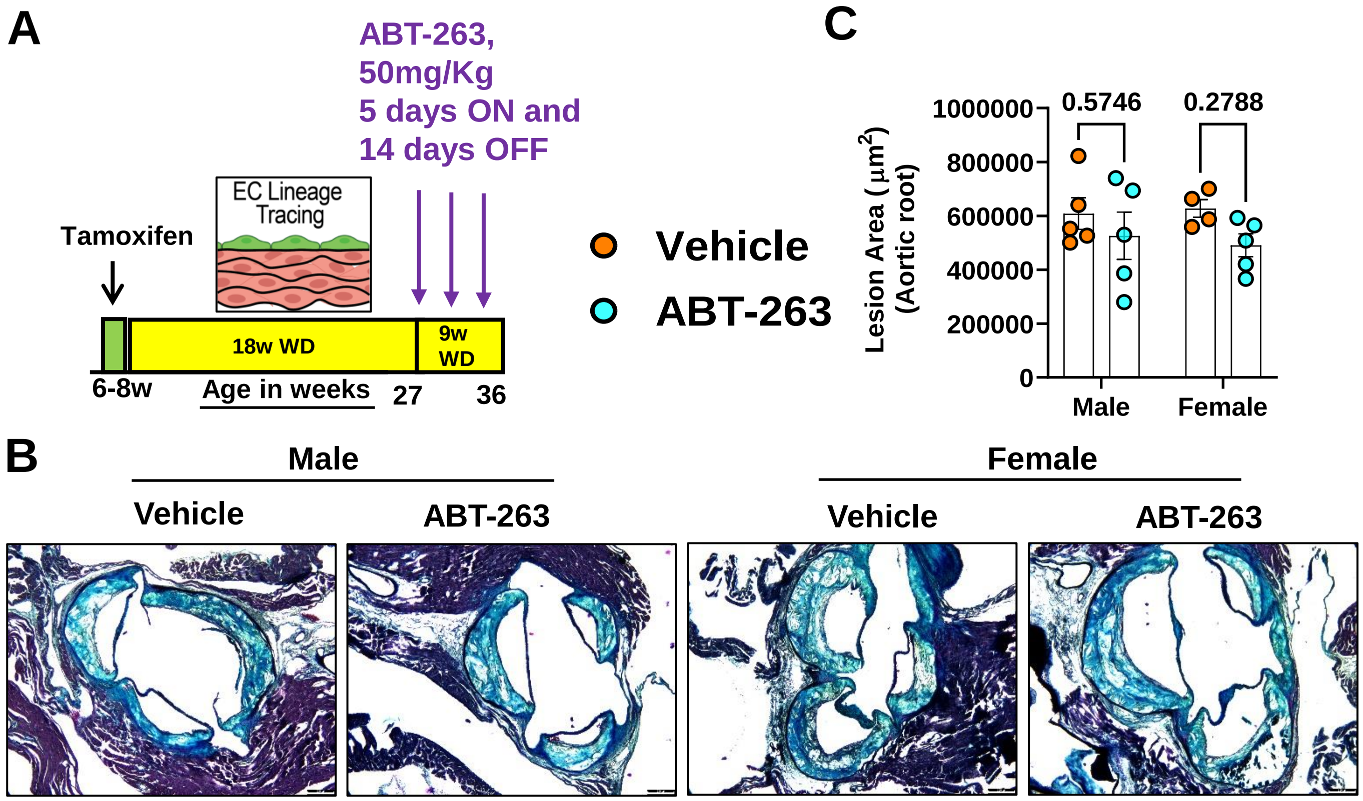

Supplemental Figure 3. A reduced dose (50mg/kg/bw) of ABT-263 treatment in *Apoe*$^{-/-}$ mice with advanced lesions did not change the aortic root lesion size. (A) Experimental design: EC-lineage tracing *Apoe*$^{-/-}$ mice were fed a WD for 18 weeks followed by 50mg/kg/bw ABT-263 treatment on WD for 9 weeks. **(B)** Representative 5x images with 100µm scale bar of MOVAT staining on Aortic root lesions. **(C)** Quantification of lesion area from E. Mann-Whitney U-tests was used for statistical analysis of panel C and Two-way ANOVA test used for F. The error bars show the standard error of the mean (SEM). Independent animals are indicated as individual dots on the graphs. The p-values are indicated on the respective graphs.

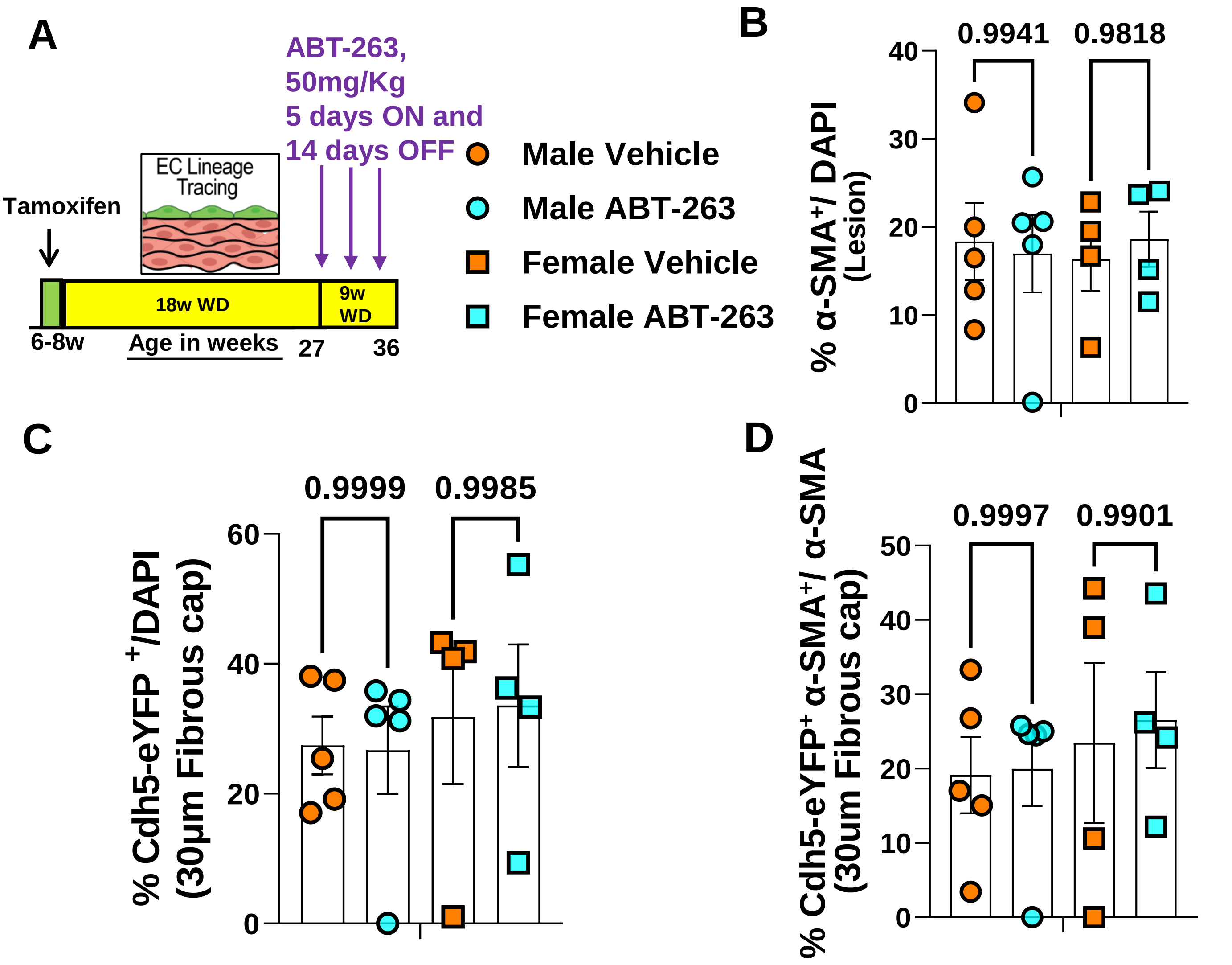

Supplemental Figure 4. A reduced dose (50mg/kg/bw) of ABT-263 treatment of *Apoe*$^{-/-}$ mice with advanced lesions did not change endothelial cell number or EC-derived α-SMA^{+} cell number in the fibrous cap. (A) Experimental design: EC-lineage tracing *Apoe*$^{-/-}$ mice were fed a WD for 18 weeks followed by 50mg/kg/bw ABT-263 treatment on WD for 9 weeks. **(B)** α-SMA^{+} cells of all (DAPI^{+}) cells in the lesion. **(C)** Endothelial cells of all (DAPI^{+}) cells in the fibrous cap. **(D)** EC-derived α-SMA^{+} cells of all α-SMA^{+} cells in the fibrous cap. A two-way ANOVA was used for statistical analysis. The error bars show the standard error of the mean (SEM). Independent animals are indicated as individual dots on the graphs. The p-values are indicated on the respective graphs.

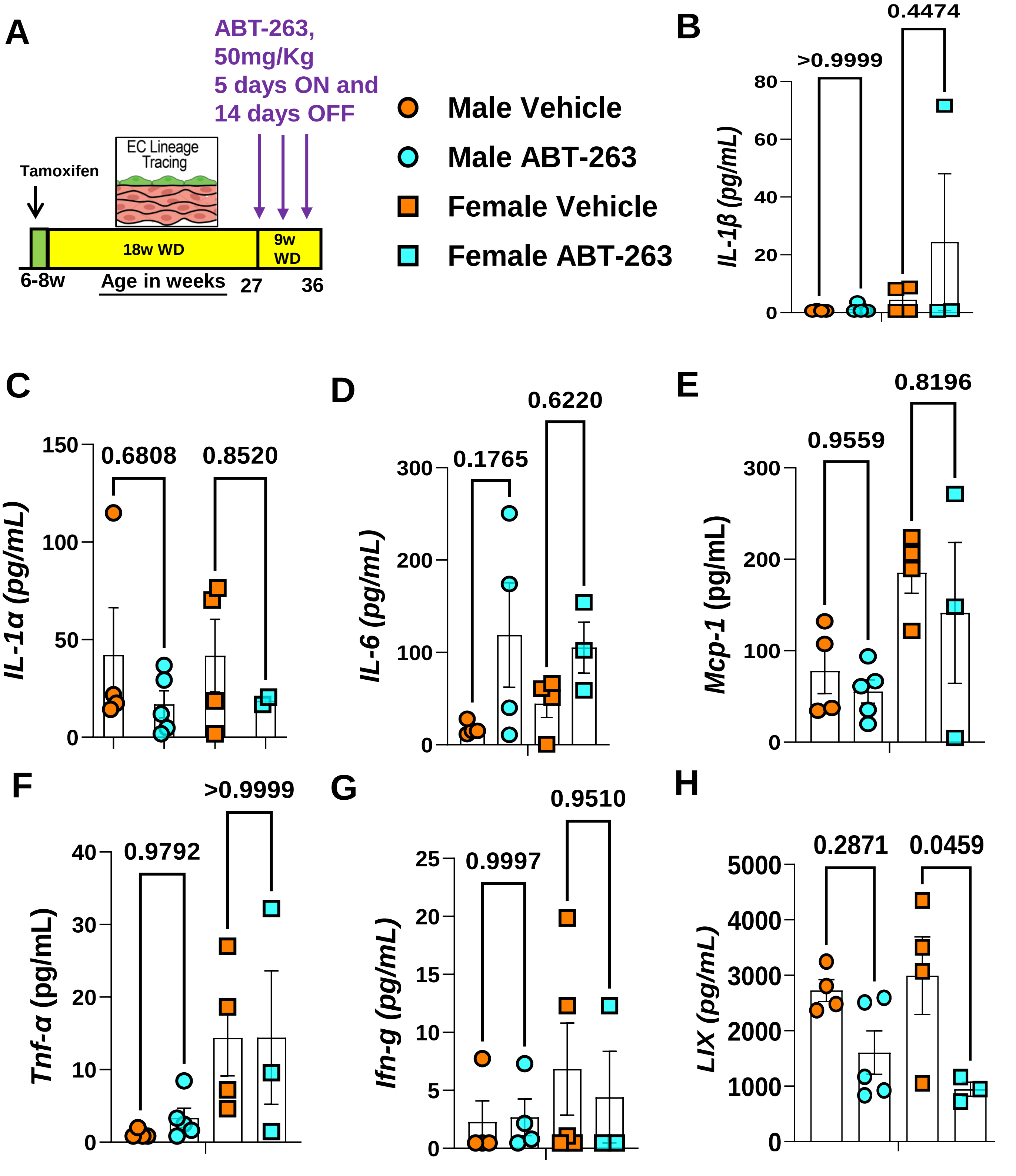

Supplemental Figure 5. Low dose (50mg/kg/bw) ABT-263 treatment of *Apoe$^{-/-}$* mice with advanced lesions was associated with reduced plasma LIX (Cxcl5), but did not reduce SASPs and cytokines including, IL-1β, IL-1α, IL-6, Mcp-1, Tnf-α, and Ifn-g. (A) Experimental design, EC-lineage tracing *Apoe$^{-/-}$* mice were fed a WD for 18 weeks followed by 50mg/kg/bw ABT-263 treatment on WD for 9 (3 cycles- 5 days ON and 14 days OFF) weeks. Plasma **(B)** IL-1β, **(C)** IL-1α, **(D)** IL-6, **(E)** Mcp-1, **(F)** Tnf-α, **(G)** Ifn-g, and **(H)** LIX. A two-way ANOVA was used for statistical analysis. The error bars show the standard error of the mean (SEM). Independent animals are indicated as individual dots on the graphs. The p-values are indicated on the respective graphs.

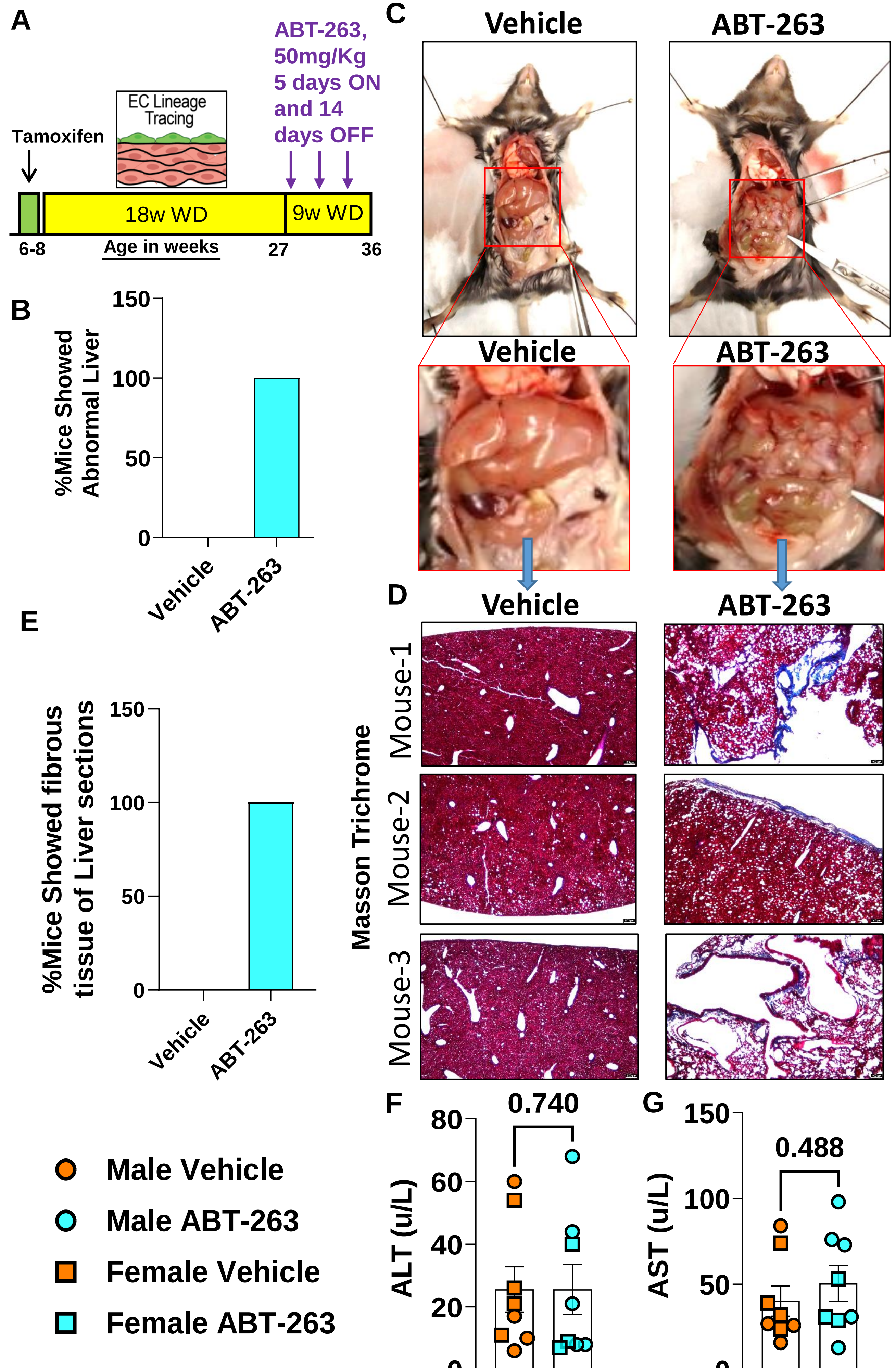

Supplemental Figure 6

Supplemental Figure 6. Low dose (50mg/kg/bw) ABT-263 treatment of *Apoe*$^{-/-}$ mice with advanced lesions was associated with increased hepatic fibrosis as shown by mason trichrome stain. (A) Experimental design: EC-lineage tracing *Apoe*$^{-/-}$ mice were fed a WD for 18 weeks followed by 50mg/kg/bw ABT-263 treatment on WD for 9 weeks. **(B)** Percentage of mice showing an abnormal liver phenotype. **(C)** Representative photographs of mice showing an abnormal liver phenotype with ABT-263 treatment. **(D)** Masson trichrome stain to detect the fibrous tissue (blue) in the liver sections from the vehicle and ABT-263 treated mice. **(E)** Percentage mice showing fibrous tissue (blue) in the liver sections from the vehicle and ABT-263 treated mice. **(F)** ALT and **(G)** AST levels measured in plasma. Mann-Whitney U-tests were used for statistical analysis in F and G, and biologically independent animals are indicated as individual dots. The error bars show the standard error of the mean (SEM). The p-values are indicated on the respective graphs.

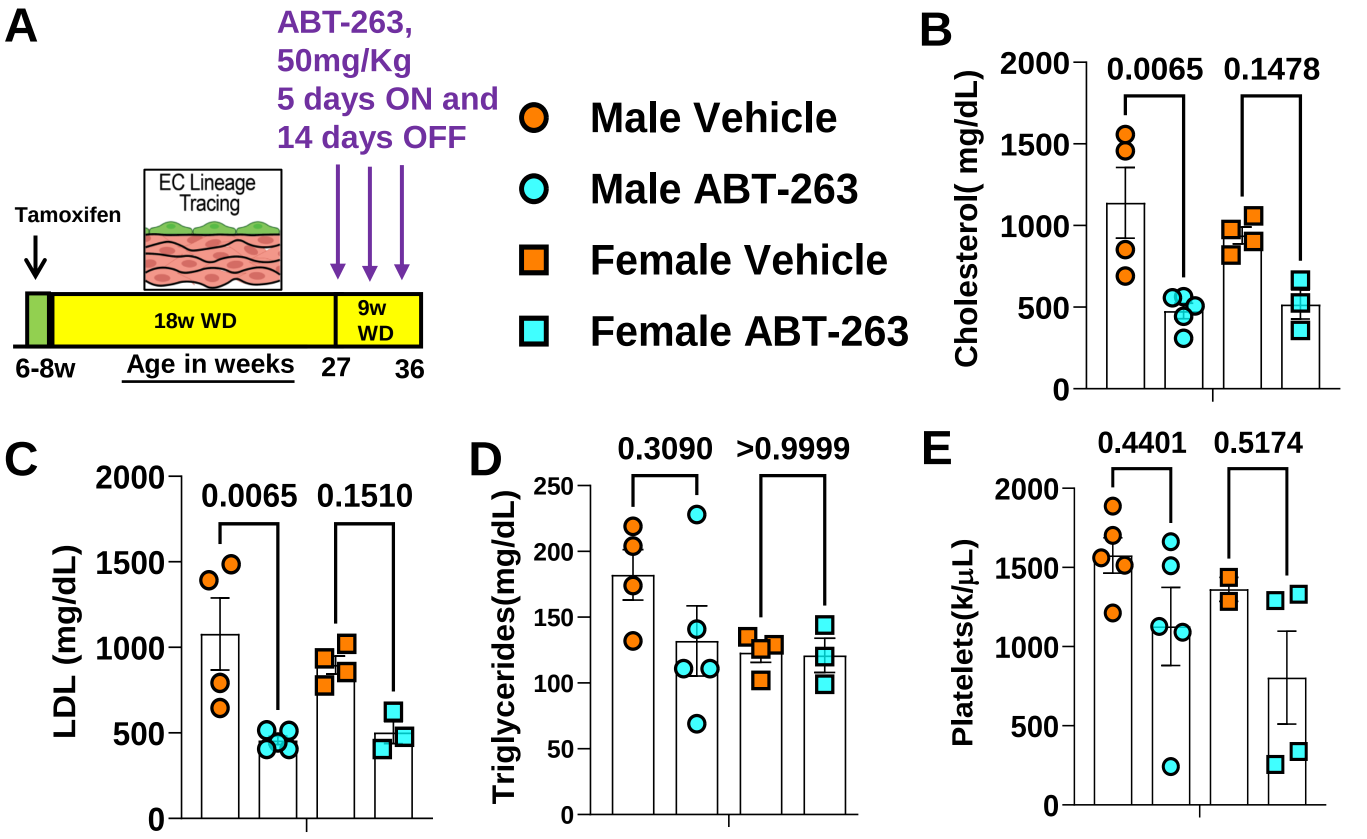

Supplemental Figure 7. Low dose (50mg/kg/bw) ABT-263 treatment of *Apoe*$^{-/-}$ mice with advanced lesions was associated with reduced plasma total cholesterol and LDL cholesterol levels in male mice. (A) Experimental design, EC-lineage tracing *Apoe*$^{-/-}$ mice were fed a WD for 18 weeks followed by 50mg/kg/bw ABT-263 treatment on WD for 9 weeks. **(B)** Plasma total cholesterol, **(C)** LDL cholesterol, **(D)** Triglycerides, and **(E)** Platelets in the blood**.** A two-way ANOVA was used for statistical analysis of B-E. Biologically independent animals are indicated as individual dots. The error bars show the standard error of the mean (SEM). The p-value is indicated on the respective graph.

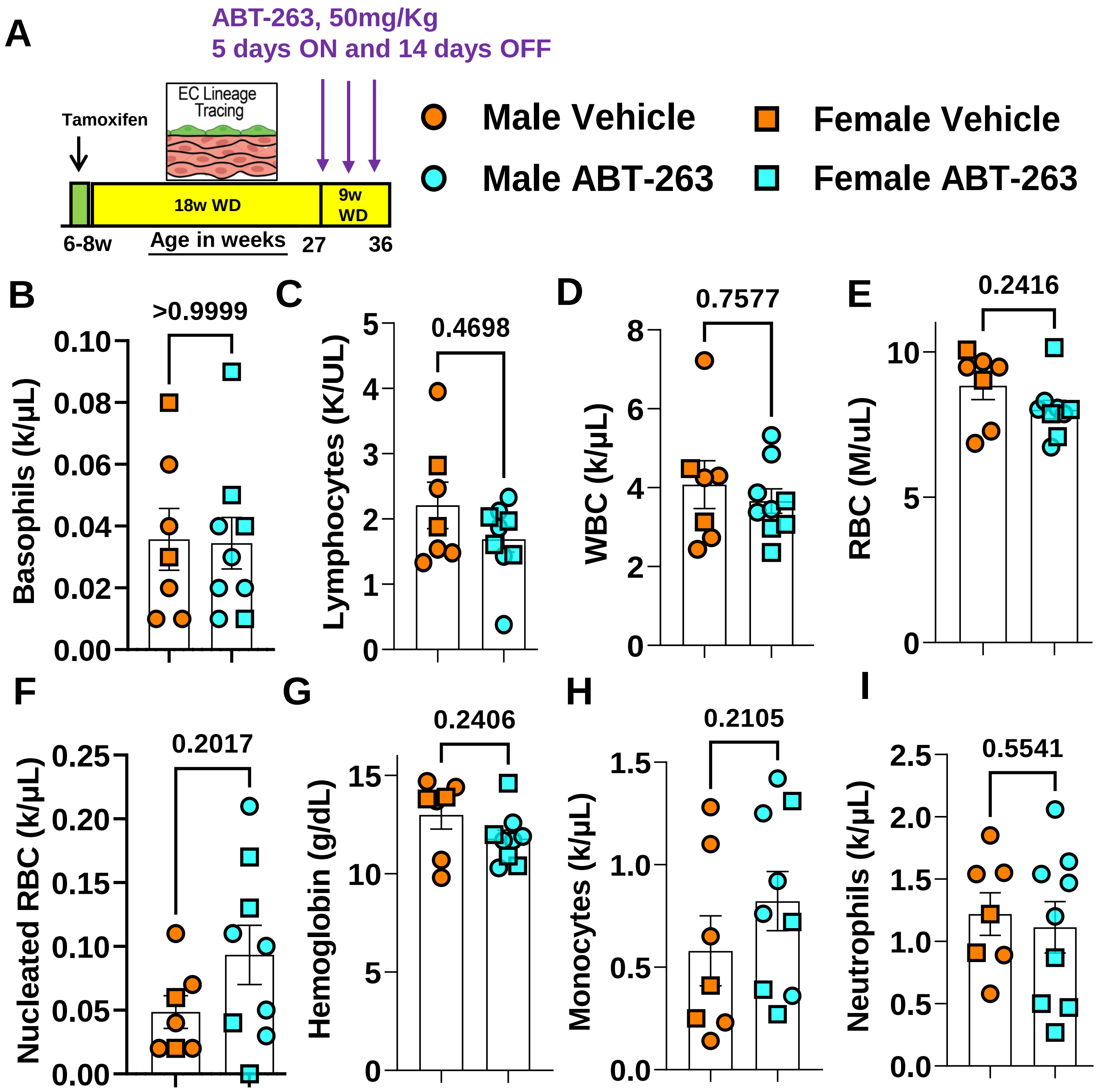

Supplemental Figure 8. Low dose (50mg/kg/bw) ABT-263 treatment of *Apoe*$^{-/-}$ mice with advanced lesions did not change cell count in the blood. (A) Experimental design, EC-lineage tracing *Apoe*$^{-/-}$ mice were fed a WD for 18 weeks followed by 50mg/kg/bw ABT-263 treatment on WD for 9 weeks. **(B)** Basophils, **(C)** Lymphocytes, **(D)** WBC, **(E)** RBC, **(F)** Nucleated RBC, **(G)** Hemoglobin, **(H)** Monocytes, and **(I)** Neutrophils in the blood. Mann-Whitney U-tests used for statistics. The error bars show the standard error of the mean (SEM). Independent animals are indicated as individual dots on the graphs. The p-values are indicated on the respective graphs.

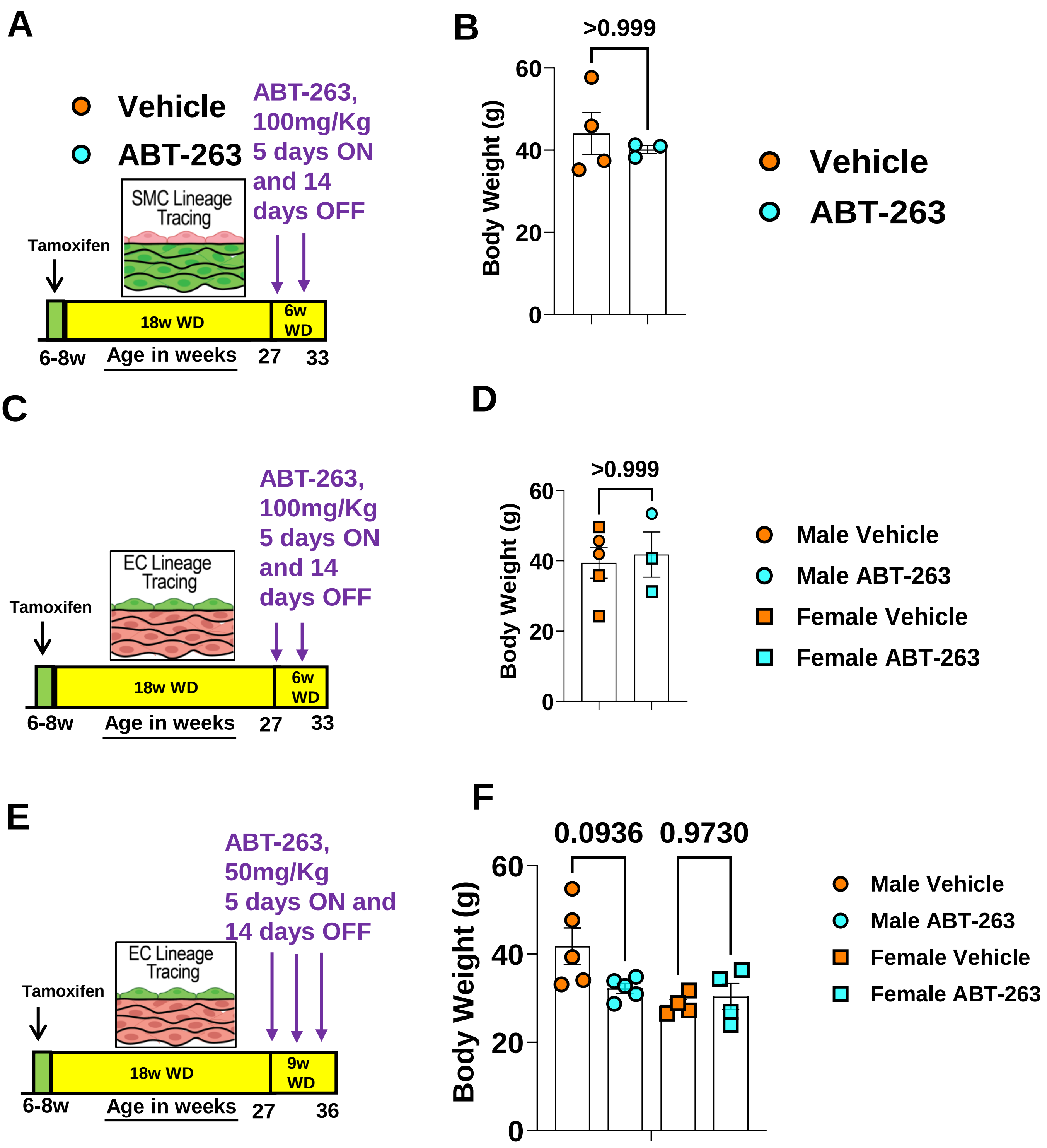

Supplemental Figure 9. ABT-263 treatment of WD-fed *Apoe$^{-/-}$* mice did not affect the body weight. (A) Experimental design, SMC-lineage tracing *Apoe$^{-/-}$* mice were fed a WD for 18 weeks followed by 100mg/kg/bw ABT-263 treatment on WD for 6 weeks. **(B)** Body weight (g), **(C)** Experimental design, EC-lineage tracing *Apoe$^{-/-}$* mice were fed a WD for 18 weeks followed by 100mg/kg/bw ABT-263 treatment on WD for 6 weeks. **(D)** Body weight (g), **(E)** Experimental design, EC-lineage tracing *Apoe$^{-/-}$* mice were fed a WD for 18 weeks followed by 50mg/kg/bw ABT-263 treatment on WD for 9 weeks. **(F)** Body weight (g). Mann-Whitney U-tests were used for statistical analysis of B and D. A two-way ANOVA was used for statistical analysis of F. The error bars show the standard error of the mean (SEM). Independent animals are indicated as individual dots on the graphs. The p-values are indicated on the respective graphs.

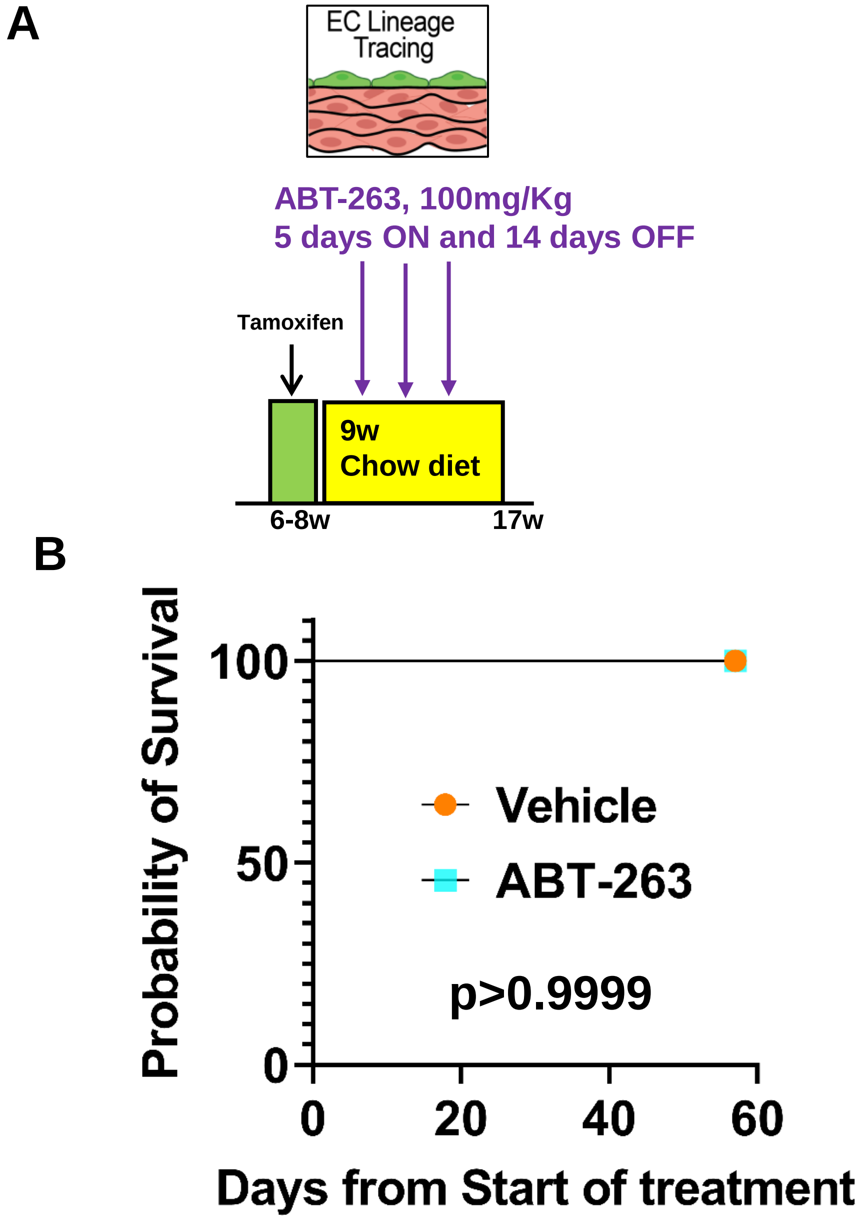

Supplemental Figure 10. EC-lineage tracing *Apoe*$^{-/-}$ mice treated with ABT-263 but fed a zero-cholesterol low fat chow diet showed 100% survival. Experimental design, 8 weeks old EC-lineage tracing *Apoe*$^{-/-}$ mice were treated with a 100mg/kg/bw ABT-263 (n=6) or vehicle (n=5) for 5 days ON and 14 days OFF cycle for three cycles. Throughout the experiment, mice were fed a standard chow diet. B. Probability of survival curve. A Mantel-Cox test was used for statistical analysis.
